# Screening of crosstalk and pyroptosis-related genes linking periodontitis and osteoporosis based on bioinformatics and machine learning

**DOI:** 10.3389/fimmu.2022.955441

**Published:** 2022-08-05

**Authors:** Jia Liu, Ding Zhang, Yu Cao, Huichao Zhang, Jianing Li, Jingyu Xu, Ling Yu, Surong Ye, Luyi Yang

**Affiliations:** ^1^ Department of Orthodontics, Hospital of Stomatology, Jilin University, Changchun, China; ^2^ Department of Spine Surgery, China-Japan Union Hospital, Jilin University, Changchun, China; ^3^ Department of Endodontics, Hospital of Stomatology, Jilin University, Changchun, China

**Keywords:** periodontitis, osteoporosis, pyroptosis, geonomics, community discovery, XGBoost, immune infiltration

## Abstract

**Background and objective:**

This study aimed to identify crosstalk genes between periodontitis (PD) and osteoporosis (OP) and potential relationships between crosstalk and pyroptosis-related genes.

**Methods:**

PD and OP datasets were downloaded from the GEO database and were performed differential expression analysis to obtain DEGs. Overlapping DEGs got crosstalk genes linking PD and OP. Pyroptosis-related genes were obtained from literature reviews. Pearson coefficients were used to calculate crosstalk and pyroptosis-related gene correlations in the PD and OP datasets. Paired genes were obtained from the intersection of correlated genes in PD and OP. PINA and STRING databases were used to conduct the crosstalk-bridge-pyroptosis genes PPI network. The clusters in which crosstalk and pyroptosis-related genes were mainly concentrated were defined as key clusters. The key clusters’ hub genes and the included paired genes were identified as key crosstalk-pyroptosis genes. Using ROC curve analysis and XGBoost screened key genes. PPI subnetwork, gene–biological process and gene-pathway networks were constructed based on key genes. In addition, immune infiltration was analyzed on the PD dataset using the CIBERSORT algorithm.

**Results:**

A total of 69 crosstalk genes were obtained. 13 paired genes and hub genes TNF and EGFR in the key clusters (cluster2, cluster8) were identified as key crosstalk-pyroptosis genes. ROC and XGBoost showed that PRKCB, GSDMD, ARMCX3, and CASP3 were more accurate in predicting disease than other key crosstalk-pyroptosis genes while better classifying properties as a whole. KEGG analysis showed that PRKCB, GSDMD, ARMCX3, and CASP3 were involved in neutrophil extracellular trap formation and MAPK signaling pathway pathways. Immune infiltration results showed that all four key genes positively correlated with plasma cells and negatively correlated with T cells follicular helper, macrophages M2, and DCs.

**Conclusion:**

This study shows a joint mechanism between PD and OP through crosstalk and pyroptosis-related genes. The key genes PRKCB, GSDMD, ARMCX3, and CASP3 are involved in the neutrophil extracellular trap formation and MAPK signaling pathway, affecting both diseases. These findings may point the way to future research.

## Introduction

Periodontitis (PD) is a common local inflammatory disease with a high prevalence, with about one in two people worldwide suffering from it (1). Interdisciplinary studies have shown that it is associated with osteoporosis(OP), a disease that is considered a systemic metabolic disorder (2). As diseases characterized by bone loss, they share common risk factors such as age, genetics, hormonal changes, smoking habits, and history of corticosteroid treatment (3). Previous studies showed that OP was associated with the loss of alveolar bone (4, 5), which could impact the progression of PD and was considered an independent risk factor for the prognosis of PD (6). Also, a recent meta-analysis pointed out that patients with PD were at a higher risk of developing OP (7). Evidence suggests that the two diseases are somehow related and can affect each other. But these reports cannot elucidate the intrinsic relationship between PD and OP.

Some scholars have recently suggested that OP may be associated with chronic systemic inflammation (8, 9). Pro-inflammatory cytokines such as IL1, IL6 and TNF-α can participate in bone remodeling by up-regulating the receptor activator of NF-kappa B ligand (RANKL). Moreover, the immune response they activate may be an essential risk factor for OP (10–12). Interestingly, periodontitis, a local oral cavity inflammation, is closely related to systemic inflammation. Recent studies have shown that bacteria or locally activated immune cells in periodontal tissue can enter extra-oral tissues during the onset and treatment of PD. They can cause inflammation and affect non-oral diseases such as cardiac metabolic diseases, autoimmune diseases, and cancer (13–17). At the same time, the effects of periodontitis and systemic status may also be mutual. Systemic diseases such as type II diabetes can increase the susceptibility to periodontitis by increasing the inflammatory burden of periodontal tissue or by regulating periodontal microorganisms (18, 19). In this context, we can boldly assume that inflammation may be an important mechanism for the link between the two diseases.

Pyroptosis is an inflammation-related programmed cell death that can be activated by inflammatory vesicles or bacterial lipopolysaccharides, resulting in cell swelling, perforation, rupture of cell membranes, and leakage of cell contents, thus provoking cell death (20). Multiple *in vitro* and *in vivo* experiments have confirmed the critical role of pyroptosis in PD (21–23), which can mediate the loss of periodontal ligament stem cells, enhance inflammation, accelerate bone destruction, and then promote the progression of PD (24). The link between OP and pyroptosis has not been confirmed experimentally. Still, the idea that pyroptosis may be the pathogenesis of OP has been recognized by several scholars (25–27), and the link between them deserves further exploration. Therefore, investigating the relationship between PD and OP combined with pyroptosis can facilitate understanding of the pathophysiological mechanisms underlying its development and guide coordinated interdisciplinary management in the clinical setting.

**Figure 1:**
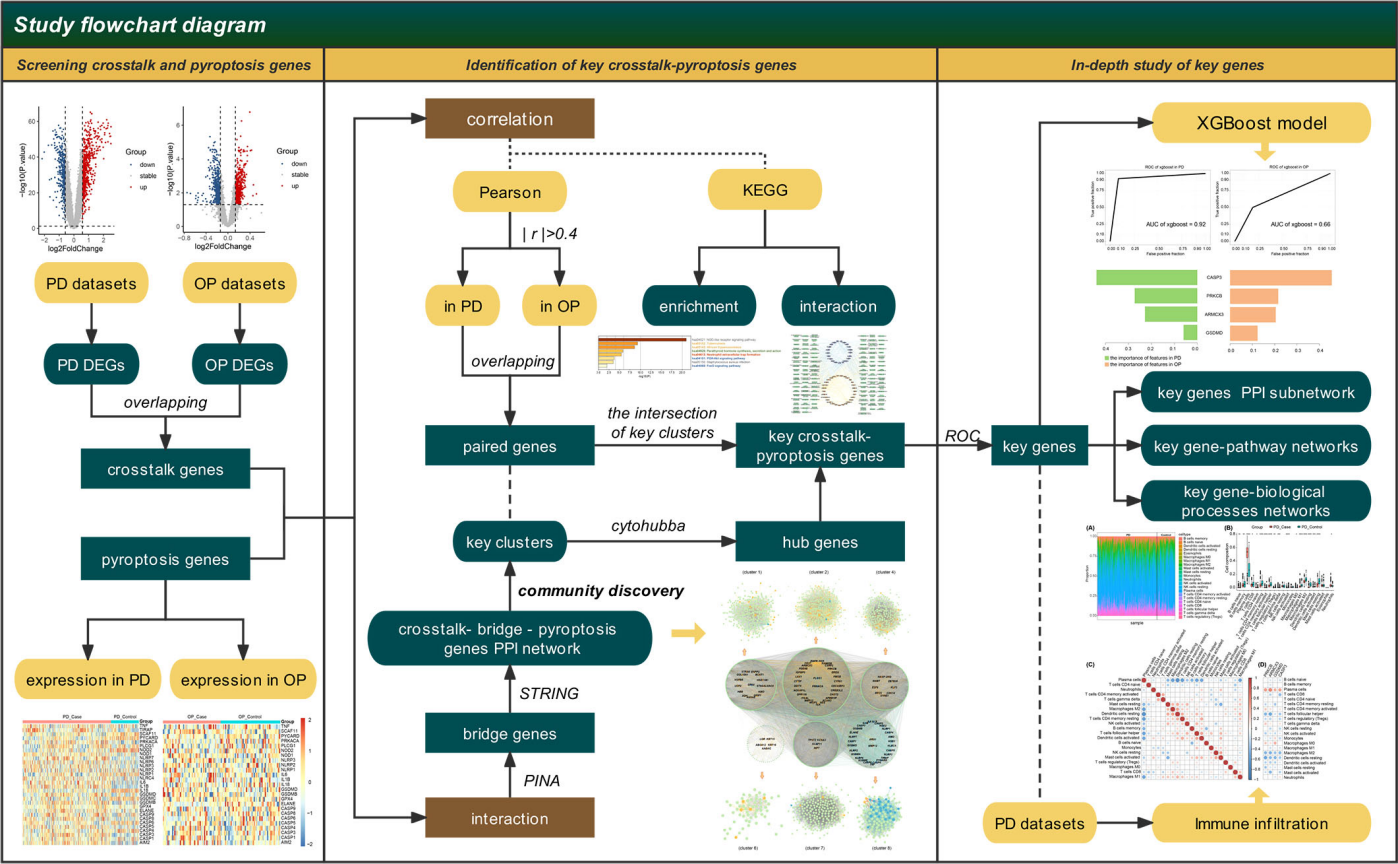
Flow-chart of datasets analysis in this paper.

We use bioinformatics to overcome the difficulty in the combined clinical study of PD and OP. By searching for the crosstalk genes between PD and OP and linking them with pyroptosis-related genes using correlation analysis and PPI network, we presume the key genes in the relationship between PD and OP and their related signal pathways, investigate the mechanism of the interaction between the two diseases, and generate additional hypotheses for clinical research problems. To better describe the interaction between genes and their effects, we present the notion of bridge genes and use immune infiltration to explain how PD and OP affect each other. (**Figure 1**)

The general hypotheses of this study are as follows. Pyroptosis is the common mechanism of PD and OP. There is a correlation between crosstalk and pyroptosis-related genes in PD and OP, and they can interact with each other through bridge genes. At least a pair of crosstalk-pyroptosis genes is  strongly correlated in both diseases. They interact with each other and influence each other’s expression to influence the development of PD and OP through some mechanisms.

## Materials and methods

### Data downloading and preprocessing

GEO (https://www.ncbi.nlm.nih.gov/geo/) is a public functional genomics database from which we obtained the PD and OP datasets. PD datasets were searched using the following keywords: “periodontitis”, “human genome” and “gingival tissue” and screened based on the following criteria: (1) each dataset contained at least 20 samples; (2) at least 10 cases and 10 controls were included; (3) the raw data or matrix files were available in the GEO database, and the experimental type was microarray. Based on such criteria, two periodontitis-related datasets (GSE16134 and GSE10334) were included in this analysis. OP datasets were searched using the following keywords: “osteoporosis”, “human genome” and “monocyte”, and the screening criteria were the same as PD. Two osteoporosis-related datasets (GSE56815 and GSE7158) were included in this analysis. The details of each GEO dataset were shown in **Table S1**.

Based on the information from the datasets, intersecting genes were obtained between each disease dataset. Regarding PD, the intersection between the genes examined in the GSE16134 and GSE10334 datasets was obtained, and the same was true for OP. The expression profiles of these intersecting genes in each dataset were obtained separately and integrated with clinical information. The ComBat method in the sva package (version 3.36.0) of R software (version 4.0.2) was used to correct the merged data in batches to reduce the bias of combining samples from different batches.

### Differential expression analysis

After batch correction, differential expression analysis (DEA) was performed on the corrected PD and OP datasets using the limma package (version 3.44.3). In PD, genes with P-values < 0.05 and | *log*_2_ (fold change) | > *log*_2_1.5 were defined as differential expressed genes (DEGs), and in OP, genes with P-values < 0.05 and | *log*_2_ (fold change) | > *log*_2_ 1.1 were defined as DEGs. The expression of DEGs was demonstrated by the ggplot2 package (version 3.3.2) and pheatmap package (version 1.0.12) with volcano plots and heatmaps.

### Functional enrichment analysis

We imported the genes to be evaluated (DEGs of PD and OP, crosstalk genes, key clusters’ genes) into the Metascape database (28) for functional enrichment analysis. GO and KEGG analyses were performed for the identified genes using P-values < 0.01, min (overlap) = 3, and min (enrichment) = 1.5 as thresholds, and heatmaps displayed the results.

### Identification of crosstalk genes

After identifying DEGs of PD and OP separately, they were imported into R software to obtain the intersection of PD DEGs and OP DEGs. These jointly dysregulated genes in PD and OP may be the keys to the links between the two, and we call them crosstalk genes.

### Correlation of crosstalk genes with pyroptosis-related genes

The literature search was carried out in PubMed for articles related to pyroptosis-related genes. A total of 370 articles were retrieved in two years. Most articles on bioinformatics define these 33 genes as pyroptosis-related genes (29–32), as detailed in **Table 1**. To investigate the role of pyroptosis in regulating the link between PD and OP, we obtained the expression profiles of crosstalk and pyroptosis-related genes in the PD and OP datasets and analyzed correlations by calculating Pearson coefficients. We used the Hmisc package (version 4.4.1) to calculate Pearson correlation coefficient (r) values and screened genes with moderate and strong correlation (P-values < 0.05 and | r | > 0.4) (33). A heatmap of the correlation between crosstalk genes and pyroptosis-related genes was also produced using the corrplot package (version 0.84). Crosstalk genes and pyroptosis-related genes correlated in PD and OP were taken to intersect, and these genes were called paired genes. These genes were studied in subsequent analyses.

**Table 1:** Pyroptosis-related genes from literature.

Genes	NCBI-GeneID	Official Full Name
AIM2	9447	absent in melanoma 2
CASP1	834	caspase 1
CASP3	836	caspase 3
CASP4	837	caspase 4
CASP5	838	caspase 5
CASP6	839	caspase 6
CASP8	841	caspase 8
CASP9	842	caspase 9
ELANE	1991	elastase, neutrophil expressed
GPX4	2879	glutathione peroxidase 4
GSDMA	284110	gasdermin A
GSDMB	55876	gasdermin B
GSDMC	56169	gasdermin C
GSDMD	79792	gasdermin D
GSDME	1687	gasdermin E
IL18	3606	interleukin 18
IL1B	3553	interleukin 1 beta
IL6	3569	interleukin 6
NLRC4	58484	NLR family CARD domain containing 4
NLRP1	22861	NLR family pyrin domain containing 1
NLRP2	55655	NLR family pyrin domain containing 2
NLRP3	114548	NLR family pyrin domain containing 3
NLRP6	171389	NLR family pyrin domain containing 6
NLRP7	199713	NLR family pyrin domain containing 7
NOD1	10392	nucleotide binding oligomerization domain containing 1
NOD2	64127	nucleotide binding oligomerization domain containing 2
PJVK	494513	pejvakin
PLCG1	5335	phospholipase C gamma 1
PRKACA	5566	protein kinase cAMP-activated catalytic subunit alpha
PYCARD	29108	PYD and CARD domain containing
SCAF11	9169	SR-related CTD associated factor 11
TIRAP	114609	TIR domain containing adaptor protein
TNF	7124	tumor necrosis factor

To complement the correlation, we used the KEGG database (34) to identify and classify the common pathways between crosstalk and pyroptosis-related genes. Using the ggplot2 package, we drew a circular barplot to display the counts of common pathways present in each class. The Cytoscape (35) (version 3.9.0) program was used to visualize the gene-common pathway network. Simultaneously, crosstalk and pyroptosis-related genes were imported into the Metascape database (28), and significant common pathways were identified using the threshold P-values < 0.01, min (overlap) = 3, min (enrichment) = 1.5.

### Construction of PPI network and community discovery analysis

Crosstalk and pyroptosis-related genes were imported into the PINA database (36) to obtain their associated genes. The genes associated with at least two crosstalk or pyroptosis-related genes were defined as bridge genes and normalized through the UniProt database (37). The bridge gene can help us better understand the context of crosstalk and pyroptosis-related genes, enrich the relationship of gene interaction, and make the following research more comprehensive and accurate. The crosstalk, bridge, and pyroptosis-related genes were imported into the STRING database (38) to obtain the PPI network. Spinglass is a clustering algorithm that focuses on minimizing outside connections while promoting within-community connections (39) and is often used to cluster networks, especially human PPI networks (40). So, we used the Spinglass community discovery function of the igraph package (version 1.2.5) to cluster the PPI network and obtain the gene clusters that influence PD and OP, where weight was set to combine score, spins to 10. The cluster network was imported into Cytoscape for visualization.

### Discovery of key clusters and key crosstalk-pyroptosis genes

The clusters in which crosstalk and pyroptosis-related genes were mainly concentrated were defined as key clusters and were imported into Cytoscape software. The TOP10 hub genes of the key clusters were identified using the cytohubba plug-in (41). The crosstalk and pyroptosis-related genes in the hub genes were selected, and these genes deserve to be explored in the shared mechanism of PD and OP. For subsequent analysis, these hub genes and the paired genes in the key clusters were defined as key crosstalk-pyroptosis genes.

### Identification of key genes

Then, we further explored the importance of key crosstalk-pyroptosis genes as a potential biomarker. For patients with PD, the method of diagnosis by the gene level of gingival tissue is not feasible, and we only study its essential effect on the disease. Receiver operating characteristic (ROC) analysis was performed on key crosstalk-pyroptosis genes in both diseases using AUC > 0.5 as the threshold (42). Among the screened genes, crosstalk genes that were up-regulated or down-regulated in both PD and OP and their correlated pyroptosis-related genes were defined as key genes.

To further investigate the role of key genes as a whole in both diseases, we defined them as a whole as the key signature. The uneven number of PD samples was solved using the smotefamily package (version 1.3.1). The PD and OP datasets were divided into training and testing sets according to the ratio of 7:3. The XGBoost package (version 1.4.1.1), a machine learning method, was used to construct the classification model with the key signature, and the importance score of each feature was viewed by “xgb.ggplot.importance” function of XGBoost package.

### GO and KEGG analysis of key genes

We extracted key genes-related subnetworks from the previous PPI network and used Cytoscape to visualize the context of key genes’ roles. To better understand the function of key genes and explore the common pathological mechanism between the two diseases, we imported key genes into the Gene Ontology (43) and KEGG database (34). We screened human biological processes and pathways containing at least two key genes. ClueGo (44) was used to fuse and cluster the obtained biological processes to explain the results better. KEGG database was used to view common pathways class and map, obtain the potential association between key genes and pathways, and between pathways and pathways.

### Immune infiltration

We used the CIBERSORT (45) algorithm in R software to obtain the immune infiltration matrix from the PD gene expression datasets. The relevant R code and documents of the CIBERSORT algorithm can be found in **Supplementary Materials** (**(Supplementary Material Data Sheet 1)**). The immune infiltration matrix data was visualized for each sample and group using the ggplot2 package. Wilcoxon test was used to compare the differences between the two groups. Then, we used the corrplot package to plot correlation heatmaps to visualize the correlation between the 22 infiltrating immune cells and the correlation between key genes and immune cells.

## Results

### Data preprocessing

**Figure 2:**
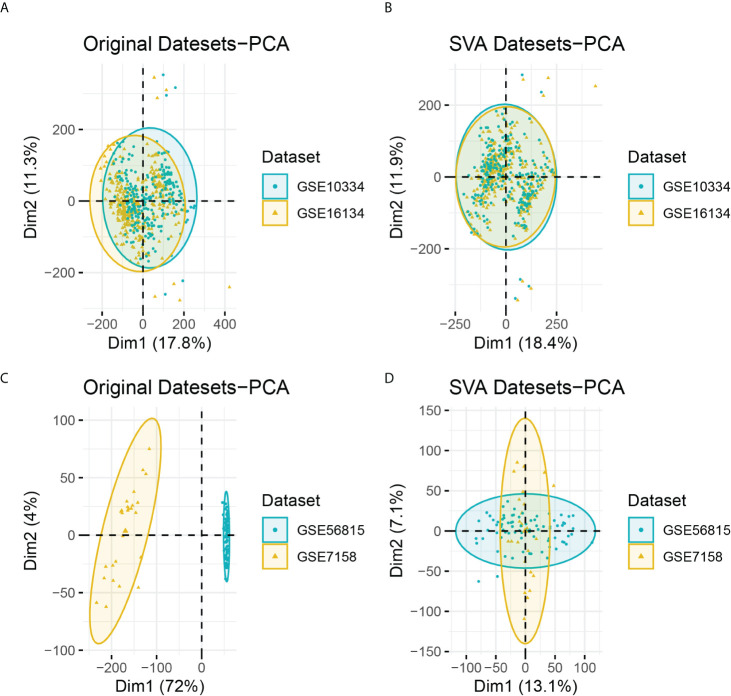
**(A)** PCA analysis results of PD datasets before batch correction; **(B)** PCA analysis results of PD datasets after batch correction; **(C)** PCA analysis results of OP datasets before batch correction; **(D)** PCA analysis results of OP datasets after batch correction.

After batch correction, the PD and OP datasets were obtained. The PD datasets contained two datasets (GSE16134 and GSE10334) with 424 case samples and 133 control samples. The OP datasets contained two datasets (GSE56815 and GSE7158), consisting of 52 case samples and 54 control samples. The differences between datasets were significantly reduced after batch correction (**Figure 2**).

### Identification and enrichment analysis of DEGs

**Figure 3:**
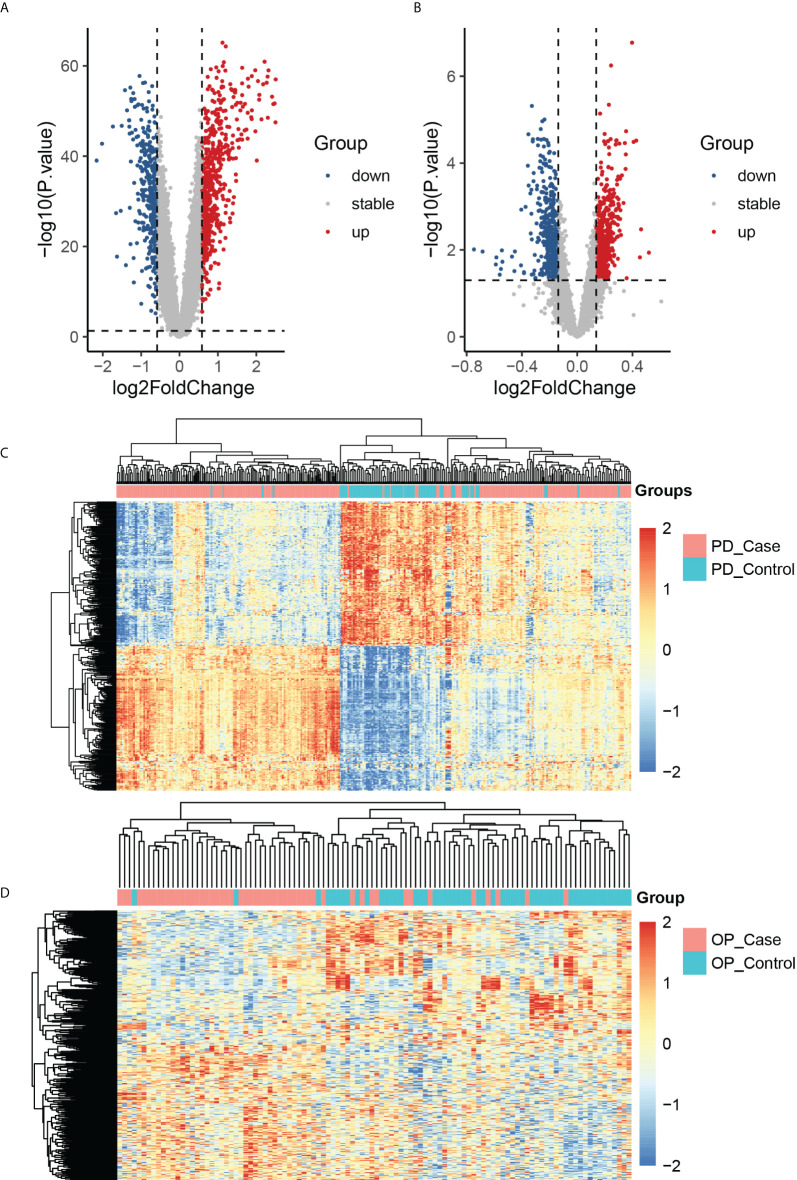
**(A)** The volcano plots of PD DEGs; **(B)** the volcano plots of OP DEGs; **(C)** the heatmap of the top 400 PD up-regulated and down-regulated genes; PD_case present the periodontitis affected gingival tissue sample, and PD_control present the periodontitis unaffected gingival tissue sample; **(D)** the heatmap of the top 400 OP up-regulated and down-regulated genes; OP_case present the samples of the monocytes from osteoporosis patient and OP_control present the samples of the monocytes from non-osteoporotic patients.

**Figure 4:**
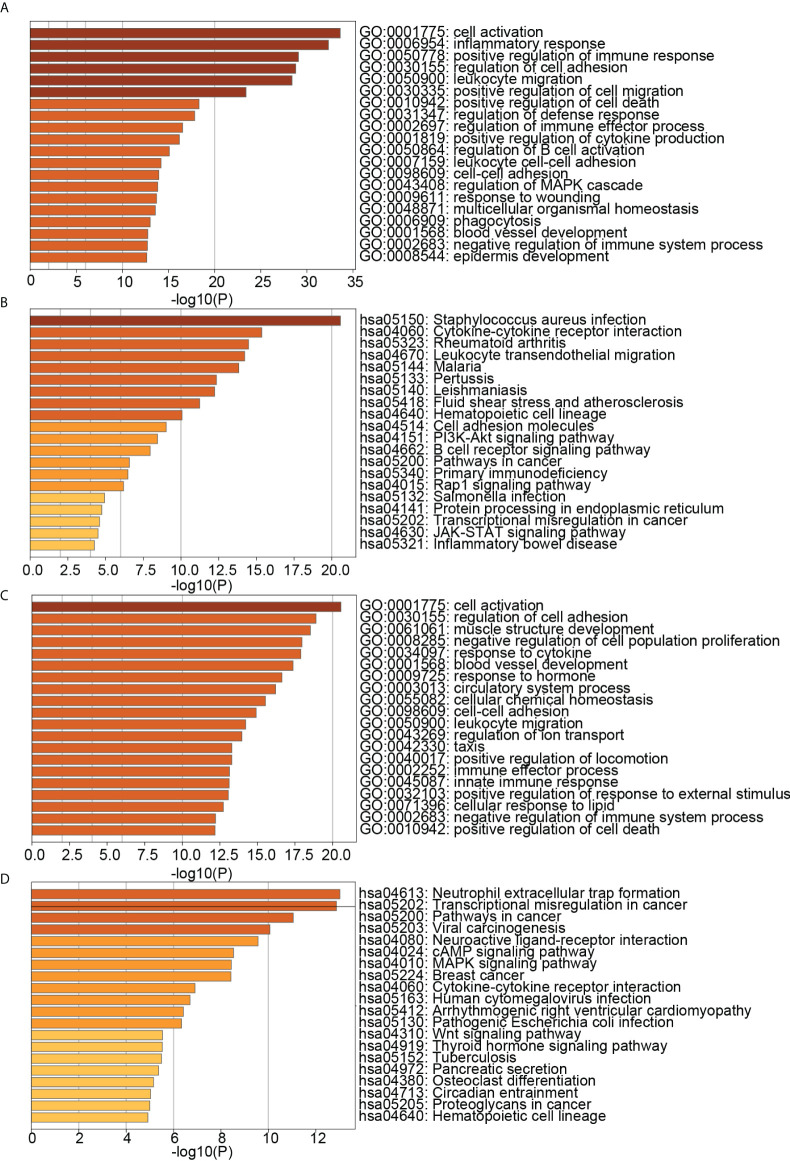
**(A)** TOP20 GO BP terms of PD DEGs; **(B)** TOP20 KEGG pathways of PD DEGs; **(C)** TOP20 GO BP terms of OP DEGs; **(D)** TOP20 KEGG pathways of OP DEGs.

We obtained DEGs of PD and OP based on differential expression analysis, with 621 up-regulated genes and 417 down-regulated genes in PD and 603 up-regulated genes and 515 down-regulated genes in OP. The expression pattern of DEGs in both diseases was depicted using a volcano plot and heatmap (**Figure 3**). GO and KEGG enrichment results showed that PD and OP were associated with immune response. In addition, both PD and OP DEGs were involved in the “positive regulation of cell death” (**Figure 4**). To further explore this result, we viewed the GO clustering results of Metascape. We found that “positive regulation of cell death” in both PD and OP is related to “positive regulation of programmed cell death” and “positive regulation of apoptotic process” (**Figure S1**). Pyroptosis is a kind of programmed cell death, which has extensive crosstalk with the process of apoptosis (46). This result confirms the hypothesis that pyroptosis is the co-development mechanism of PD and OP.

### Identification and enrichment analysis of crosstalk genes

**Figure 5:**
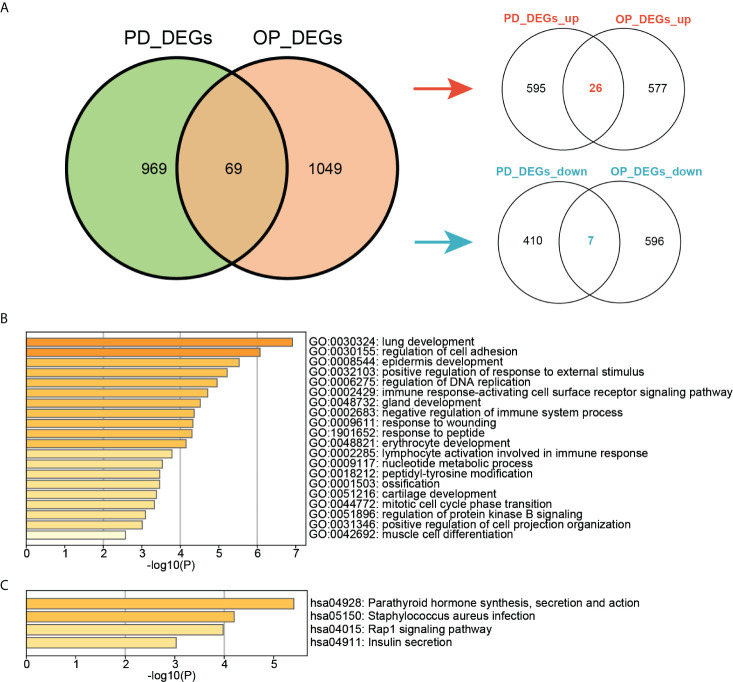
**(A)** Venn diagram of the intersection of PD DEGs and OP DEGs; **(B)**TOP20 GO BP terms of crosstalk genes; **(C)** TOP4 KEGG pathways of crosstalk genes.

The intersection of DEGs in PD and OP revealed 69 crosstalk genes, 26 of which were up-regulated and 7 of which were down-regulated in both PD and OP. These 33 genes were used for screening key genes (**Figure 5A**). Although the expression of other 36 genes in PD and OP did not exhibit the same trend, their role in the association between PD and OP cannot be completely discounted. We included them in the next experiment to determine the relationship of PD and OP, but we don’t think they play a crucial role. **Figure 5B** showed that crosstalk genes were mainly enriched in several biological processes, for example, immune response-activating cell surface receptor signaling pathway, negative regulation of immune system process, lymphocyte activation involved in immune response, ossification, and cartilage development. **Figure 5C** shows that crosstalk genes were mainly enriched in several pathways, Parathyroid hormone synthesis, secretion and action, Staphylococcus aureus infection, Rap1 signaling pathway, and Insulin secretion.

### Correlation analysis of crosstalk genes with pyroptosis-related genes

**Figure 6:**
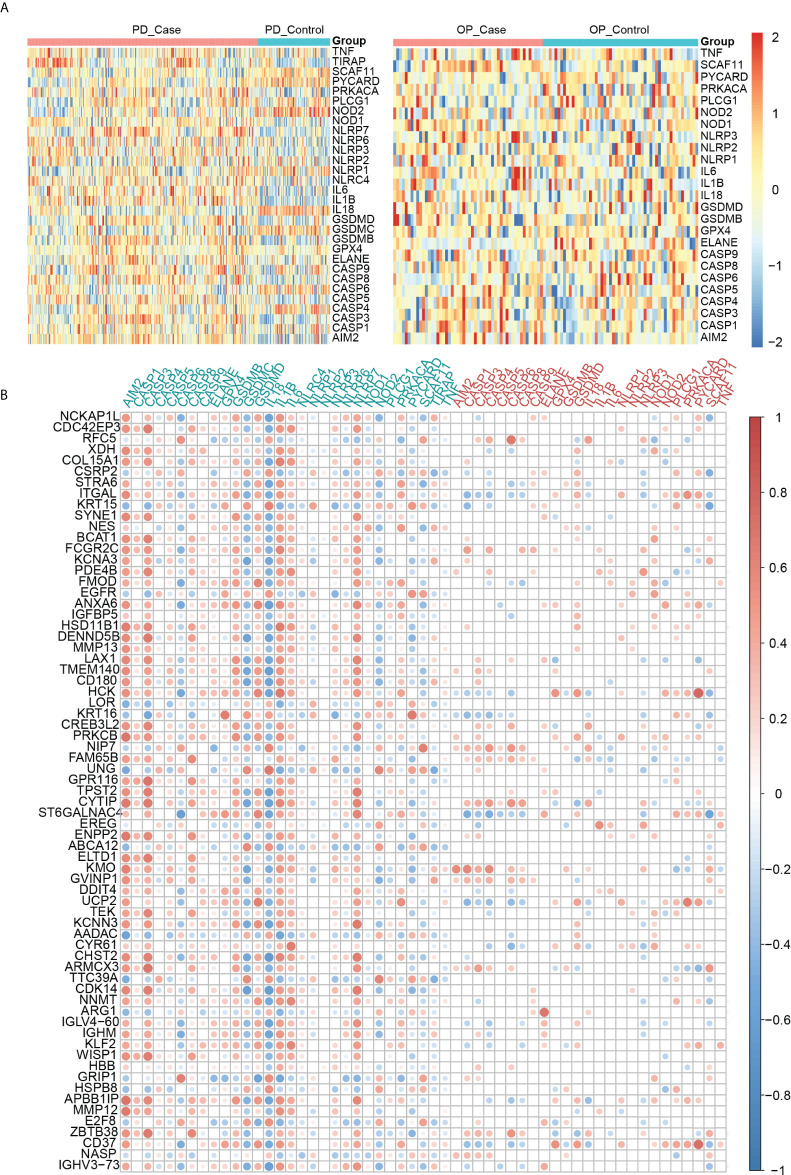
**(A)** Heatmap of pyroptosis-related genes expression in PD and OP samples; **(B)** Heatmap of the correlation between crosstalk and pyroptosis-related genes. Abscissa labels were the pyroptosis-related genes in different disease samples; blue represents PD, and red represents OP. The ordinate labels were crosstalk genes.

Another vital part of our study was the pyroptosis-related genes. We observed their expression in both diseases using heatmaps (**Figure 6A**). In PD, 25 pyroptosis-related genes differed significantly in the PD group compared to the control group (P-value < 0.05). Among them IL6, IL1B and IL18 were PD DEGs. Four pyroptosis-related genes differed significantly in the OP group compared to the control group (P-value < 0.05). Among these, AIM2, ELANE, and SCAF11 were OP DEGs. In addition, the expression of other pyroptosis-related genes was not differential (**Table S2**). Pearson correlation coefficients analyzed the correlations between 33 pyroptosis-related genes and 69 crosstalk genes (**Figure 6B**). A total of 413 pairs of associated crosstalk-pyroptosis genes were identified in PD, 57 pairs in OP (P-values < 0.05, | r | > 0.4), and 17 pairs of associated genes presented in both diseases (including ten crosstalk genes and eight pyroptosis-related genes). These 18 genes were defined as paired genes to be further studied (**Table S3**).

**Figure 7:**
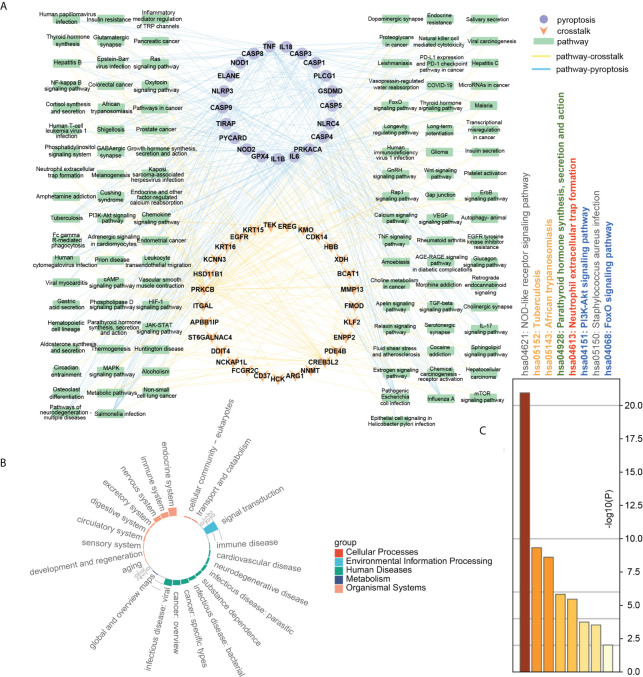
**(A)** Gene-common pathway network; **(B)** the circle barplot of common pathways’ class counts; **(C)** TOP8 pathways of crosstalk and pyroptosis-related genes. Gray: not a common pathway. Common pathway: yellow: human diseases; green: endocrine system; red: immune system; blue: signal transduction pathway.

To further explore the correlation between crosstalk and pyroptosis-related genes, we used the KEGG and Metascape databases for pathway analysis. The results of the KEGG database analysis showed that 99 genes participated in 225 pathways, including 111 common pathways involved in crosstalk and pyroptosis-related genes. **Figure 7B** revealed that common pathways were primarily involved in “signal transduction”, endocrine system” and “immune system” and were closely related to “infectious diseases” and “cancer”. To further understand the relationship between crosstalk and pyroptosis-related genes, we drew a gene-common pathway network consisting of 30 crosstalk genes, 22 pyroptosis-related genes, 111 common pathways, and 482 edges connecting pathways and genes (**Figure 7A**). It showed that PRKCB and four pyroptosis-related genes (TIRAP, IL1B, PLCG1, TNF) commonly affected NF-kappa B signaling pathway; four crosstalk genes (EGFR, EREG, PRKCB, TEK) together with four pyroptosis-related genes (IL1B, PRKACA, TNF, CASP3) regulated MAPK signaling pathway. On the other hand, Metascape pathway enrichment analysis identified eight significant pathways, six of which were common pathways (**Figure 7C**). By examining the class of significant common pathways, we determined that one pathway was associated with the immune system, one with the endocrine system, two with infectious diseases, and two with signal transduction. Gene-common pathway network analysis and enrichment analysis revealed that immune system and endocrine system pathways not only connect crosstalk and pyroptosis-related genes but also can be affected by them.

### Construction and subcluster analysis of PPI network

**Figure 8:**
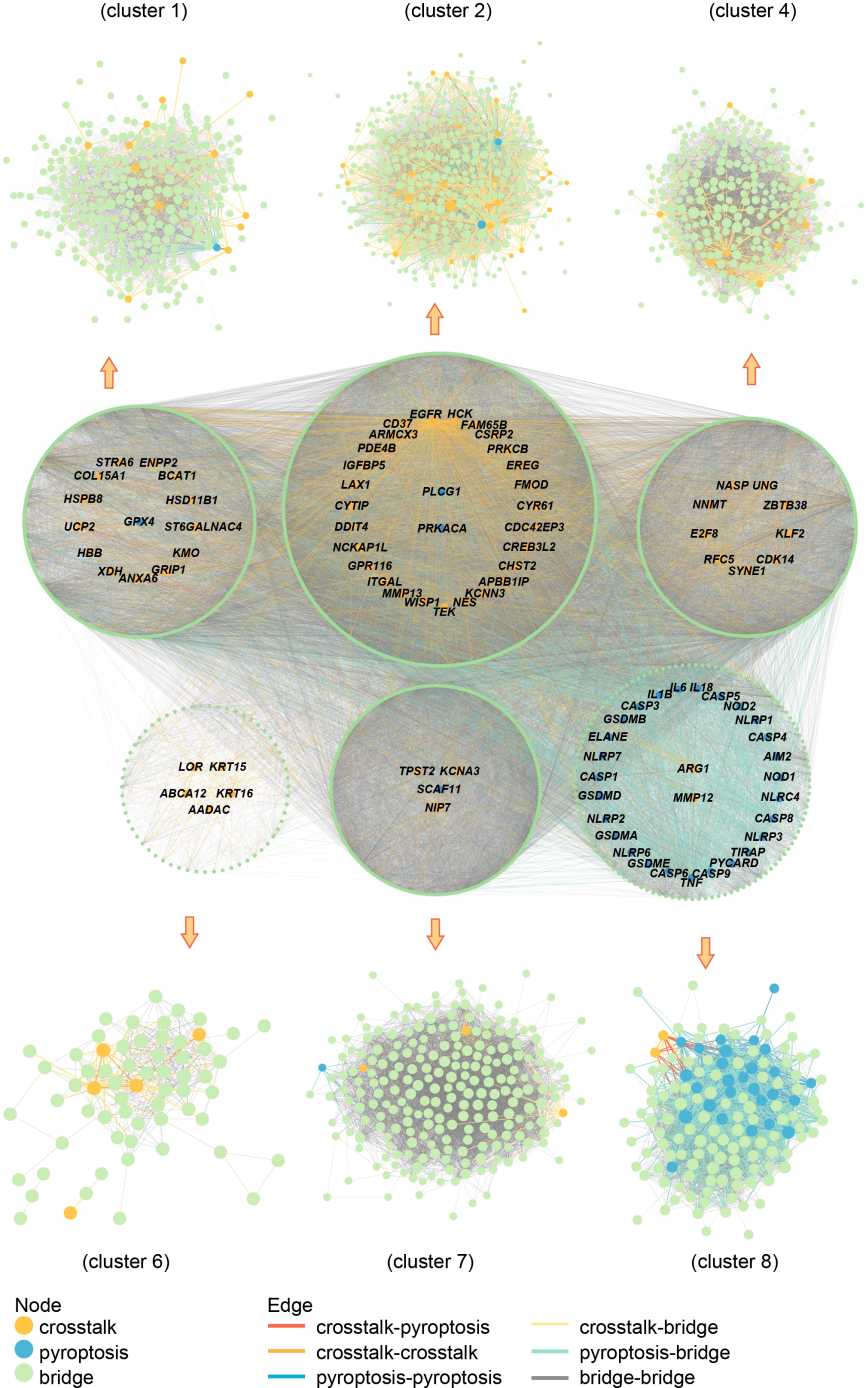
Community discovery clustering network. The node size increases with the degree value.

The crosstalk and pyroptosis-related genes were imported into the PINA database, screened, and normalized to obtain 1409 genes, including 32 pyroptosis-related genes, 64 crosstalk genes, and 1313 bridge genes (**Table S4**). The PPI network of crosstalk, bridge, and pyroptosis-related genes, including 1381 nodes, and 40855 edges, was obtained using the STRING database (**Table S5**). The genes among them were divided into 9 clusters using the Spinglass community discovery algorithm. These nine gene clusters had a stable and closely related internal structure. **Table S6** displays the number of genes present in each cluster. After removing the clusters with less than 20 genes, 6 clusters were selected (**Figure 8**). Density is an evaluation standard used to measure the density of interconnection edges between nodes in a network (47). The density of these six clusters was shown in **Table S7**. The density of cluster 1 was the lowest (0.07492169), but it was also higher than that of the PPI network before clustering (0.04287483). This result proved that the partition result of the multilevel algorithm was reliable. The genes in each cluster were closely linked and could act as a whole, playing different roles in the diseases. Remarkably, crosstalk and pyroptosis-related genes were mainly concentrated in clusters 2 and 8, so we defined clusters 2 and 8 as key clusters. The genes contained in these two clusters may be involved in the shared mechanism of both diseases.

**Figure 9:**
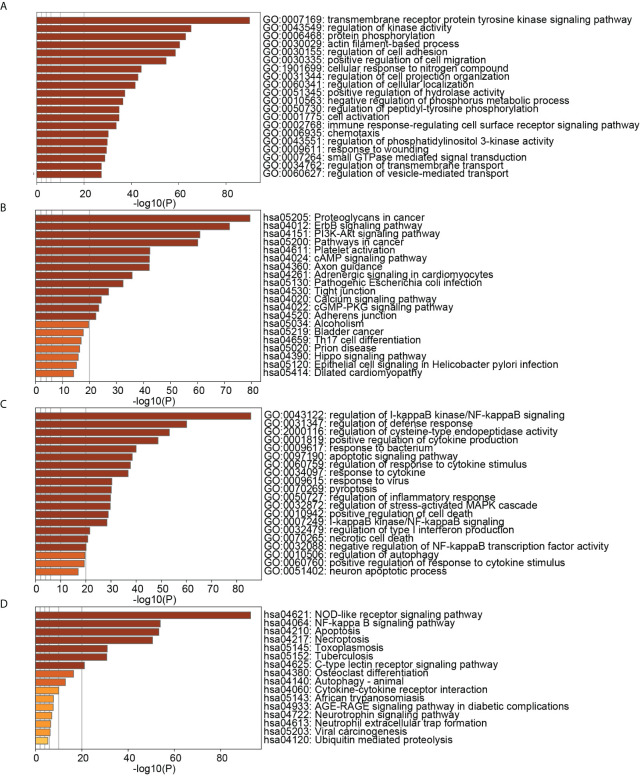
**(A)** TOP20 GO BP terms of cluster2 genes; **(B)** TOP20 KEGG pathways of cluster2 genes; **(C)** TOP20 GO BP terms of cluster8 genes; **(D)** TOP20 KEGG pathways of cluster8 genes.

**Figure 9** showed that the biological processes of cluster 2 were mainly enriched in transmembrane receptor protein tyrosine kinase signaling pathway, regulation of kinase activity, protein phosphorylation, actin filament-based process, regulation of cell adhesion, positive regulation of cell migration, etc. The pathways of cluster 2 were mainly enriched in Proteoglycans in cancer, ErbB signaling pathway, PI3K-Akt signaling pathway, Pathways in cancer, Platelet activation, and cAMP signaling pathway. The biological processes of cluster 8 were mainly enriched in regulation of I-kappaB kinase/NF-kappaB signaling, regulation of defense response, regulation of cysteine-type endopeptidase activity, positive regulation of cytokine production, response to the bacterium, apoptotic signaling pathway. The pathways of cluster 8 were mainly enriched in the NOD-like receptor signaling pathway, NF-kappa B signaling pathway, Apoptosis, Necroptosis, Toxoplasmosis, and Tuberculosis. These results suggested that cluster 2 may affect PD and OP by regulating cellular kinase activation, cell migration, etc. Cluster 8 may affect PD and OP by regulating cellular defense responses, programmed cell death, etc.

### Identification of key crosstalk-pyroptosis genes

**Figure 10:**
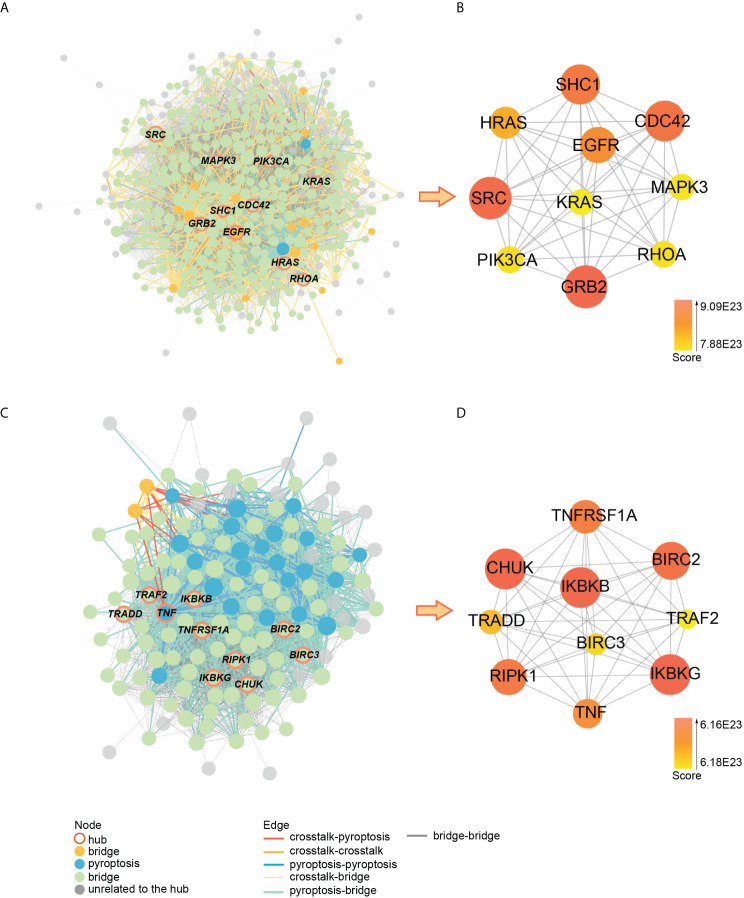
**(A)** PPI network of cluster2 genes; **(B)** the relationship between hub genes in cluster2; **(C)** PPI network of cluster8 genes; **(D)** the relationship between hub genes in cluster8.

Using cytohubba, we obtained the top 10 hub genes in clusters 2 and 8 (**Figure 10**). The pyroptosis-related gene TNF, together with the crosstalk gene EGFR as hub genes, played an essential role in clusters 2 and 8, which may be the key to the link between PD and OP. Meanwhile, we took the intersection of clusters 2 and 8 genes with paired genes, and we got seven genes PRKACA, ARMCX3, CD37, CYTIP, HCK, ITGAL, PRKCB for cluster 2 and six genes CASP3, GSDMD, IL18, CASP8, AIM2, CASP6 for cluster 8. These results showed that these thirteen genes, closely associated with PD and OP, also had an important position in the PPI network. We defined these thirteen genes and hub genes as key crosstalk-pyroptosis genes, which influenced the link between PD and OP from the perspective of the PPI network and Pearson correlation, respectively.

### In-depth study of key crosstalk-pyroptosis genes

**Figure 11:**
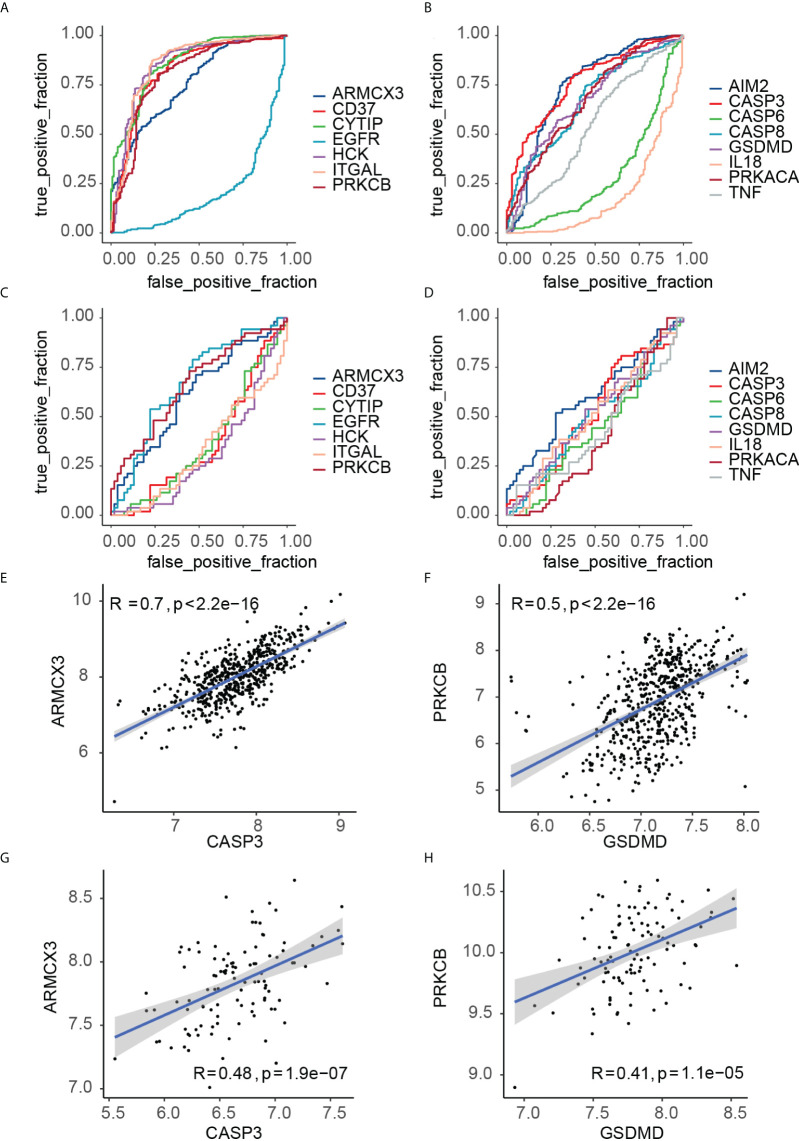
**(A)** ROC curve analysis of key crosstalk genes in PD samples; **(B)** ROC curve analysis of key pyroptosis genes in PD samples; **(C)** ROC curve analysis of key crosstalk genes in OP samples; **(D)** ROC curve analysis of key pyroptosis genes in OP samples. **(E)** correlation between ARMCX3 and CASP3 in PD samples; **(F)** correlation between PRKCB and GSDMD in PD samples; **(G)** correlation between ARMCX3 and CASP3 in OP samples; **(H)** correlation between PRKCB and GSDMD in OP samples;.

The ROC curves of the 15 key crosstalk-pyroptosis genes in PD and OP were shown separately (**Figures 11A–D**). The crosstalk genes (ARMCX3, PRKCB) and the pyroptosis genes (AIM2, CASP3, GSDMD) with AUC > 0.5 in both diseases were selected (**Table S8**). We chose PRKCB and GSDMD, ARMCX3 and CASP3 as key genes because the crosstalk genes ARMCX3 and PRKCB were up-regulated in both diseases, and there was a link between PRKCB and GSDMD, ARMCX3 and CASP3 (**Figures 11E–H**).

**Figure 12:**
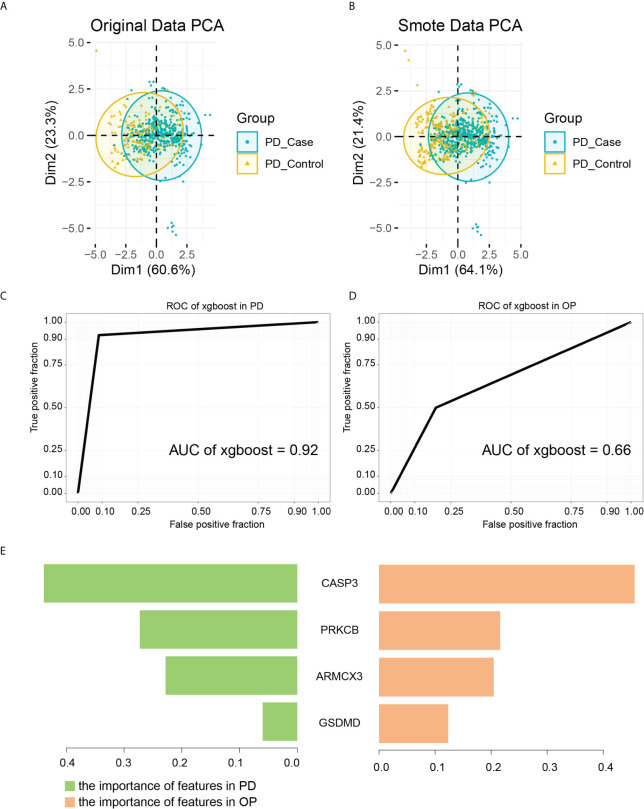
**(A)** PCA analysis results of PD original data; **(B)** PCA analysis results of PD smote data; **(C)** the ROC of XGBoost in PD samples. The AUC is 0.92; **(D)** the ROC of XGBoost in OP samples. The AUC is 0.66; **(E)** the importance of features in PD and OP samples.

We defined key genes as the key signature to further study their influence as a whole in PD and OP. The Smotefamily package was used to solve the PD sample heterogeneity problem and performed with PCA (**Figures 12A, B**). Using the method of XGBoost, the model was established according to the key signature (**Figures 12C, D**). We found that the signature showed good classification performance in PD (AUC=0.92). The classification efficiency of OP is slightly poor (AUC=0.66). However, the model’s classification was more significant than that individual gene, whether in PD or OP. These four genes had a substantial role in the progression of the two diseases and were interconnected.

As a tree-based algorithm, the XGBoost model can provide each feature’s importance score and rank it. In the single-tree model, the importance score of each feature was calculated by the improved performance metric of the partition point. The greater the promotion of a feature to the split point, the more important the feature is (48). By checking the importance score of each feature, we can understand the influence of each feature on the model. In our model, CASP3 and PRKCB were the most important features in both diseases (**Figure 12E**), and they were from different gene pairs, reinforcing the importance of both pairs of genes.

### Analysis of biological processes and pathways of key genes

**Figure 13:**
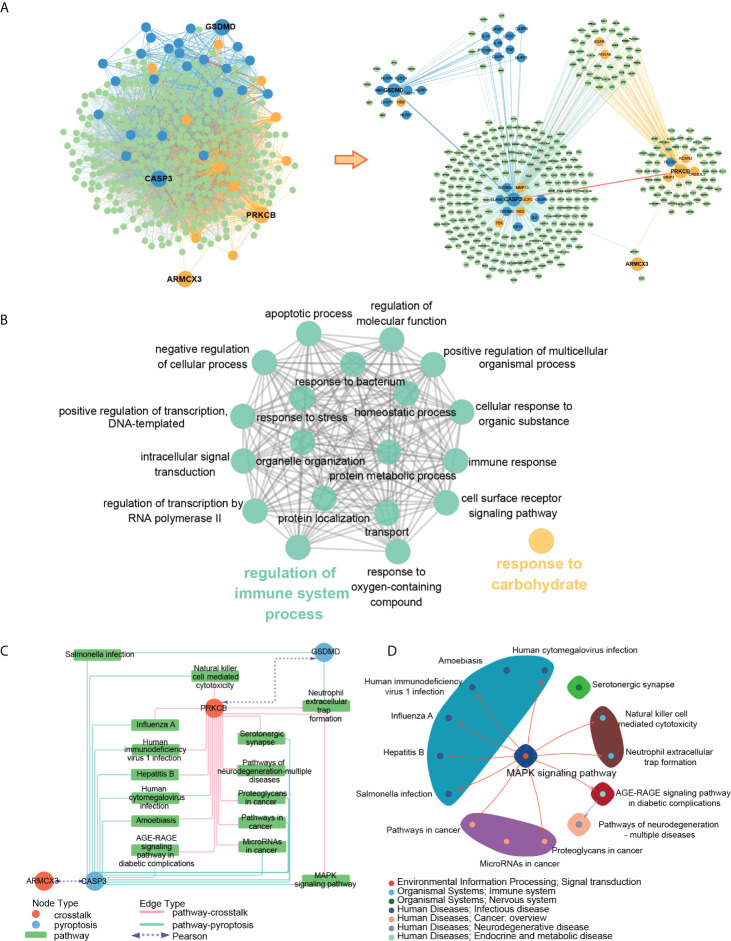
**(A)** PPI subnetwork of key genes; **(B)** ClueGO fusion and cluster result of biological processes involved in key genes; **(C)** key gene-pathway networks; **(D)** the relation of key pathways.

**Figure 13A** showed the PPI subnetwork in which key genes participated. To better observe the relationship between key genes, we extracted a subnetwork composed of key genes and the genes directly interacting with them. We found that ARMCX3 can interact with PRKCB and CASP3 through SPTAN1, PRKCB can interact with CASP3 through EGFR, ANXA6 and other genes, and CASP3 can interact with GSDMD through IL18, IL1B and other genes.

To better understand the function of key genes, we imported the key genes into the Gene Ontology database and obtained 82 biological processes containing at least two key genes (**Table S9**). The ClueGO results showed that 82 biological processes were fused into 20 and divided into two groups according to the relationship of GO terms, in which “regulation of immune system” contained most of the biological processes (**Figure 13B**).

To better explore the concrete mechanism of key genes in PD and OP, we imported the key genes into the KEGG database and obtained 15 pathways containing at least two key genes after screening (**Figure 13C**). By reviewing the information of the screened pathways (**Table S10**), we found that 6 of the 15 pathways were related to infectious diseases, and 2 pathways (Neutrophil extracellular trap formation and Natural killer cell-mediated cytotoxicity) belonged to the immune system. This result was consistent with the previous enrichment analysis of DEGs and crosstalk genes, thereby providing further evidence for the relationship between PD, OP, and the immune system. Moreover, “Neutrophil extracellular trap formation” was most significant in the pathway enrichment of OP DEGs (**Figure 4D**), and it may be an essential pathway for our study. To further explore the relation of the key pathways, we check the maps of key pathways (**Figure 13D**). MAPK signaling pathway was involved in the majority of the key pathways, including neutrophil extracellular trap formation. It may be another critical pathway for us to explore the mechanism.

### Immune infiltration

**Figure 14:**
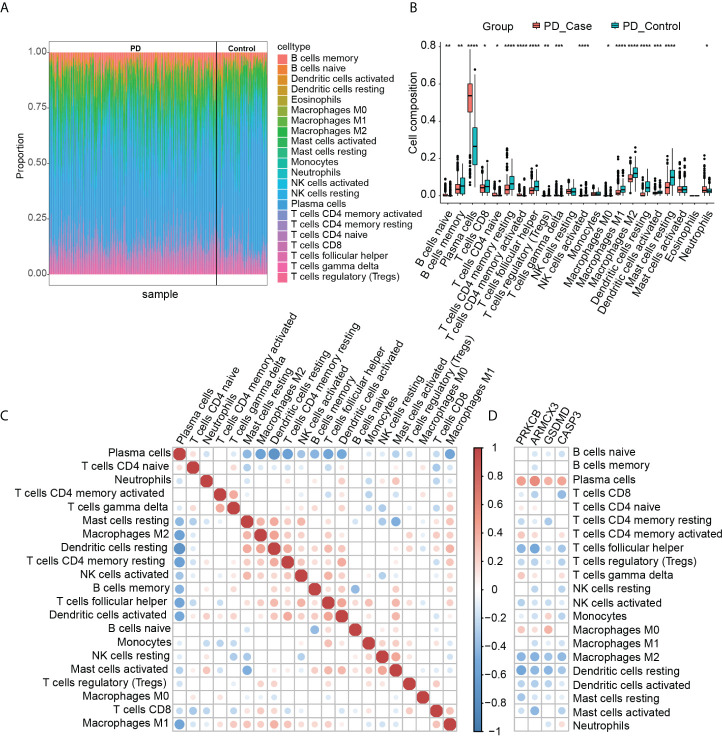
**(A)** Immune infiltration of each sample; **(B)** comparison of immune cells between the two groups (*P-value < 0.05, **P-value < 0.01, ***P-value < 0.001, ****P-value < 0.0001); **(C)** correlation between immune cells; **(D)** correlation between immune cells and key genes.

By analyzing the pathways involved in key genes, we found that PD and OP seem to be closely related to the immune system, so we used the CIBERSORT algorithm to analyze the immune infiltration of PD datasets to explore further the role of immune cells in PD and the effect of key genes on it. **Figure 14A** summarizes the results obtained from 424 control and 133 PD patients. We found significant differences between PD and normal gingival tissue in 18 immune cell subpopulations (P-values < 0.05). Compared to normal tissue, PD gingival tissue usually contained a higher proportion of naive B cells, plasma cells, T cells CD4 naive, T cells CD4 memory activated, T cells gamma delta, macrophages M0, and neutrophils (**Figure 14B**). **Figure 14C** showed the correlation between individual immune cells, and it can be seen that plasma cells have a strong negative correlation with other immune cells (P-values < 0.05). The correlation of the four key genes with each immune cell was shown in **Figure 14D**. All four genes had a positive correlation with plasma cells and a negative correlation with T cells follicular helper, M2-type macrophages, and DCs.

## Discussion

This study investigated the relationship between PD, OP, and pyroptosis through bioinformatics, community discovery, and machine learning. Two crosstalk genes (ARMCX3, PRKCB) and two pyroptosis-related genes (CASP3, GSDMD) were selected as the key genes to explain the link between PD and OP, and it was speculated that pathways such as the MAPK signaling pathway, neutrophil extracellular trap formation may be the potential common mechanism of PD and OP. The following discussion will be based on these results.

As two significant diseases affecting bone health, PD and OP are intricately linked. A growing body of evidence suggests that these two diseases share multiple risk factors and may affect each other (49). Recently, the immune response is becoming a trend in the common pathogenesis of PD and OP research (50). Enhanced cytokines and elevated inflammatory response can exacerbate bone resorption, inhibit bone formation, and lead to bone loss. Hajishengallis et al. found that locally activated lymphocytes in PD may lead to inflammation, bone marrow alterations, and immunosuppression, leading to diseases of other systems such as the skeleton (51). In this study, crosstalk genes of PD and OP mostly participated in biological processes such as cartilage development, and ossification, confirming the link between PD and OP in bone remodeling. It was noteworthy that crosstalk genes play a significant role in lymphocyte activation involved in immune response, matching those observed in earlier studies and laying the foundation for a common immune-inflammatory mechanism in both diseases.

As a pro-inflammatory cell death, pyroptosis is closely related to many diseases, such as infectious diseases and cancer (52). It plays an important role in bone destruction and promoting inflammation (53–55), and its relationship with the pathological mechanism of PD and OP has been fully studied in this context (25, 56–58). Unfortunately, in our study, pyroptosis is not the main process involved in PD and OP DEGs, but its related process of programmed cell death and apoptosis is very significant. Recent studies have found that programmed cell death is a complex mechanism. Cell death modalities are independent of each other, but they also have extensive crosstalk (46). Interestingly, as a major participant in apoptosis, the caspases family also plays an important role in pyroptosis (59), and the boundary between apoptosis and pyroptosis seems to be blurred. Therefore, the potential role of pyroptosis and its related genes in PD and OP is worth exploring.

Interestingly, in our study, the expression of most of the pyroptosis-related genes in PD cases was significantly different from that in the control group. But in OP, the results were not very optimistic; only four genes were significantly different from the control group. Current studies have shown that the role of pyroptosis in OP is mostly related to osteoclasts (58). In this study, the OP samples chosen for analysis was circulating monocytes. Perhaps because monocytes are only the precursor cells of osteoclasts (60), not osteoclasts themselves, most pyroptosis-related genes are not differentially expressed. However, it is worth noting that AIM2, which is differentially expressed in OP, can participate in the formation of inflammasomes, and its inhibitor can reduce osteoclast differentiation in bone marrow cells (61). And AIM2 is highly expressed in OP circulating monocytes, which may promote osteoclast differentiation and affect OP.

A total of four genes were identified in our study to elucidate the links between PD and OP. PRKCB, also known as PKCB, PKCβ, is a serine and threonine-specific protein kinase. Its family members can participate in various cellular signal transduction pathways closely related to infectious diseases and cancer (62) and play an essential role in bone cell metabolism (63). The current study found that PRKCB can regulate the activity of RANKL, participate in osteoclast formation and regulate its activity (64, 65). Interestingly, the polymorphism of PRKCB was found to be significantly associated with a lower level of 25 (OH) D affecting bone health (66), proving the close relationship between PRKCB and bone remodeling and confirming its potential role in PD and OP.

GSDMD, a member of the Gasdermin family, can be cleaved by inflammation-activated caspase-1 and lipopolysaccharide-activated caspase-4, -5, and -11 (67), forming plasma membrane pores at the C-terminus and releasing IL1β and IL18 (68), which is considered to be a key event in the onset of pyroptosis. Recently, it was found that GSDMD and GSDME are the only GSDM readily expressed in the bone microenvironment (69). It promotes osteoclast differentiation (70) and inhibits osteoblast proliferation and differentiation through the pyroptosis mechanism (71). Since the disorder of bone homeostasis is a common pathological process in PD and OP, the potential role of GSDMD in PD and OP seems credible. In addition, the GSDM family is associated with genetic susceptibility to inflammatory diseases such as inflammatory bowel disease and controls the balance between pyroptosis and apoptosis together with the caspase family (72).

Our other key gene, CASP3, is a member of caspases, a family of cysteine-dependent endoproteases. The family proteins are critical participants in programmed cell death such as apoptosis and pyroptosis (73) and play a vital role in inflammatory diseases (74). Its genetic polymorphism is associated with various tumor risks (75). Among them, CASP3 is considered to be the executioner of apoptosis, and its role in PD and OP has been studied repeatedly in this context (76–78). Moreover, recent studies have found that it can activate GSDME-mediated pyroptosis (79). Surprisingly, a study showed that CASP3 gene polymorphism was associated with susceptibility to periodontal disease (80), which further supported the effect of CASP3 on PD.

ARMCX3 is another important crosstalk gene in our study, which regulates mitochondrial aggregation and transport (81). Its family genes are involved in many biological processes, such as mitochondrial transport, nuclear transport and transcriptional activation (82), and are closely related to various tumors (83, 84). However, research about ARMCX3 in PD and OP still faces a severe shortage. The relationship between ARMCX3, PD and OP is worthy of further discussion.

Interestingly, the four key genes not only work alone but also have connections with each other. Activation of PRKCB can induce CASP3-mediated apoptosis (85). CASP3 can cascade with GSDME to activate pyroptosis in the absence of GSDMD. Recent studies have found that CASP3 can also cleave GSDMD-related proteins and induce secondary pyroptosis (86). Experiments have not confirmed the direct link between ARMCX3 and other genes, but our PPI network analysis shows that it may interact with CASP3 and PRKCB through SPTAN1.

Our analysis indicates that the combined effects of the four key genes on PD and OP are more significant than a single gene. The mechanism by which they operate has become another critical issue in our research. Pathway analysis revealed that CASP3 and PRKCB participate in the MAPK signaling pathway. MAPK is a highly conserved module mainly involved in cell proliferation and differentiation. It has been demonstrated to play a role in inflammation, osteoblast, and osteoclast regulation (87, 88). In our study, it also plays a significant role in the formation of neutrophil extracellular traps (NETs) in which GSDMD and PRKCB are involved. MAPK signal pathway can be activated by ROS (reactive oxygen species) and ERK to facilitate the release of NETs (89). Mitochondria play a crucial role in forming NETs (90), and a link between ARMCX3, which regulates mitochondrial aggregation, and NETs appears plausible. It appears to constitute an inferred mechanism involving the key gene, the MAPK signaling pathway, and the formation of NETs.

NETs are a body’s defense response to extracellular pathogens and have been found in various inflammation-related diseases. In PD, NETs have been shown to accelerate the formation of periodontal pockets by promoting the inflammatory response (91) and may increase local soft and hard tissue destruction (92). Furthermore, a combined clinical study showed that periodontal treatment could lead to significant changes in circulating NETs, affecting clinical manifestations of other diseases (93). The formation of NETs was the most significant in the OP DEGs biological process in our study, suggesting that NETs also play an essential role in OP. It is then reasonable to hypothesize that local inflammation caused by PD can lead to changes in circulating NETs levels, which may impact the OP.

In addition, in the key gene pathway analysis, the pathways involved in infectious diseases accounted for half, which attracted our attention. Infectious diseases, whether viral, bacterial or parasitic, can cause innate and adaptive immune responses, resulting in increased recruitment and activation of neutrophils and NK cells and arousing specific B cell and T cell immunity, leading to host inflammatory response (94–99). There is a similar mechanism in PD and OP. Existing studies have proved that oral hygiene activities can cause periodontal bacteria such as porphyromonas gingivalis to enter the blood and cause bacteremia, resulting in changes in neutrophils, plasma cells and other immune cells (100–102). For OP, the results are intriguing. Changes in hormone levels and age place OP patients in a long-term state of chronic inflammation and may cause changes in NK cells, monocytes, etc. (103, 104).

The pathway analysis results indicate that the immune system is the primary mechanism linking PD and OP. That bone homeostasis is also closely tied to the alterations in immune cells (104). Changes in immune cells appear to play a greater role in the relation between PD and OP. A recent meta-analysis denoted that the changes in local immune cells in PD may influence circulating immune cells, thereby influencing other diseases (105). Interestingly, in our study, the amounts of B cells, T cells and neutrophils, closely related to bone remodeling, are higher in the gingival tissue of PD than those in normal tissue (106). This suggests that their changes could be a reason why PD affects OP. This result was consistent but not identical to the earlier study by Li et al. (107). Activated memory CD4+ T cells, T cells gamma delta, and macrophage M0 were also significantly higher in our study, probably due to various methods of combining datasets. After examining immune infiltration and discovering similarities between the two datasets, Li et al. merged the two datasets. While in this study, we considered the batch effects between different datasets and analyzed the immune infiltration after the batch effects were processed.

Key genes are essential, co-varied genes in PD and OP, and their effects on immune cells may be the underlying mechanism for the link between PD and OP. Our study showed that the crosstalk genes (PRKCB, ARMCX3), which were highly expressed in both PD and OP, and their positively related pyroptosis-related genes (CASP3, GSDMD) might increase the content of plasma cells and reduce the content of M2-type macrophages and DCs. As a type of B cell, plasma cells have been implicated in regulating bone homeostasis in previous studies (108). Their elevation may tip the homeostasis balance toward bone resorption (109). M2 are anti-inflammatory macrophages. They can secrete proteins such as BMP-2 to induce osteoblast differentiation, and when the ratio of M2-type cells decreases, the osteogenic process may be inhibited (110). This means the key genes expression of circulating immune cells in OP patients increases, and the plasma cells and M2 cells in gingival tissue will follow the change. It will disrupt bone homeostasis, accelerate bone resorption, and inhibit bone formation, which may increase the risk of PD or aggravate the PD symptoms in OP patients.

In summary, according to the current research results, we can assume that there are three potential relationships between PD and OP: (I) Local inflammation in patients with periodontitis can cause changes in the contents of B cells, T cells and neutrophils in the circulatory system, which in turn affect the bone homeostasis of OP. (ii) The state of systemic inflammation and the differential expression of key genes in OP patients can lead to changes in the content of immune cells in gingival tissue, increasing the risk of PD or aggravating pre-existing PD symptoms. (iii) Key genes may affect PD and OP by affecting programmed cell death and bone metabolism, participating in MAPK signal pathway and inducing the production of NETs. However, these are still speculations, and the specific mechanism needs more experiments to verify.

### Strengths and limitations

The study is the first to consider the role of pyroptosis-related genes in PD and OP and to explore the links between them using bioinformatics. Applying novel tools such as machine learning and community discovery makes this study more comprehensive and novel. In previous studies, the analysis of PD and OP was limited to cross-sectional phenomena. We constructed a complete combination of crosstalk genes, pyroptosis-related genes, and their associated pathways, filling the gap in previous studies on the mechanism and broadening new ideas for future studies. However, several limitations exist. OP is a systemic disease, and the ethical and present experimental conditions allow us to use only peripheral blood samples, and the limitation of sampling may influence the results. Since the situation may be different in patients of PD and OP, individual differences should also be accounted for. In addition, this study fundamentally enables bioinformatics analysis using computer technology, which still needs to be verified experimentally.

## Conclusion

This study shows a common mechanism between PD and OP through crosstalk and pyroptosis-related genes, supporting the close interrelationship between PD and OP. The key genes PRKCB, GSDMD, ARMCX3, and CASP3, by acting on the MAPK signaling pathway, participate in the neutrophil extracellular trap formation process and affect both diseases. They may serve as a potential biomarker to guide future research in the field.

## Data availability statement

Publicly available datasets were analyzed in this study. This data can be found at GEO data repository (https://www.ncbi.nlm.nih.gov/geo/) and include the accession numbers: GSE16134, GSE10334, GSE56815 and GSE7158.

## Author contributions

LYY and JL conceived and designed the study. DZ performed data analysis and data interpretation. YC, HZ, and JNL conducted bioinformatics and statistical analyses. JX, LY, and SY generated the figures and tables. DZ and JL wrote the first draft, and LYY conceptualized and revised the manuscript. All authors contributed to the article and approved the submitted version.

## Funding

This study was funded by the Jilin Provincial Health and Health Technology Innovation Program “Study on the correlation of crown and root morphology of anterior teeth based on genetic algorithm” (2020J050).

## Acknowledgments

We thank Dr. Zeng Jianming (University of Macau) and all members of his bioinformatics team-biointerns-for generously sharing their experience and code.

## Conflict of interest

The authors declare that the research was conducted in the absence of any commercial or financial relationships that could be construed as a potential conflict of interest.

## Publisher’s note

All claims expressed in this article are solely those of the authors and do not necessarily represent those of their affiliated organizations, or those of the publisher, the editors and the reviewers. Any product that may be evaluated in this article, or claim that may be made by its manufacturer, is not guaranteed or endorsed by the publisher.

## Supplementary material

Supplementay Data Sheet 1 The relevant R code and documents of the CIBERSORT algorithm.

Supplementary Figure 1 The GO clustering results of Metascape.

Supplementary Table 1 The information of datasets.

Supplementary Table 2 Result of pyroptosis-related genes of differentially expressed analysis.

Supplementary Table 3 Paired genes in detail.

Supplementary Table 4 Filtered PINA result in detail.

Supplementary Table 5 SRTING result in detail.

Supplementary Table 6 The clusters information of community discovery.

Supplementary Table 7 The density of each clusters.

Supplementary Table 8 AUC of key crosstalk and pyroptosis-related genes in PD and OP.

Supplementary Table 9 The key genes counts and details of the biological processes involved in key genes.

Supplementary Table 10 The information of the pathways involved in key genes.

## Abbreviations

PD, periodontitis; OP, osteoporosis; DEG, differential expressed gene; FC, fold change; FDR, false discovery rate; XGBoost, Xtreme Gradient Boosting; GO, Gene Ontology; BP, biological process; KEGG, Kyoto Encyclopedia of Genes and Genomes; STRING, Search Tool for the Retrieval of Interacting Genes; PPI, protein-protein interaction; ROC, receiver-operating characteristics; AUC, the area under the curve; NETs, neutrophil extracellular traps; ROS, reactive oxygen species.

## References

[1] Del Pinto R Pietropaoli D Munoz-Aguilera E D'Aiuto F Czesnikiewicz-Guzik M Monaco A. Periodontitis and hypertension: Is the association causal? High Blood Pressure Cardiovasc Prev (2020) 27:281–9. doi: 10.1007/s40292-020-00392-z 32500479

[2] Wang C-W McCauley LK. Osteoporosis and periodontitis. Curr Osteoporos Rep (2016) 14:284–91. doi: 10.1007/s11914-016-0330-3 PMC5654540 27696284

[3] Hong S-J Yang B-E Yoo D-M Kim S-J Choi H-G Byun S-H. Analysis of the relationship between periodontitis and osteoporosis/fractures: a cross-sectional study. BMC Oral Health (2021) 21:125. doi: 10.1186/s12903-021-01496-1 33731091 PMC7968237

[4] Tezal M Wactawski-Wende J Grossi SG Ho AW Dunford R Genco RJ. The relationship between bone mineral density and periodontitis in postmenopausal women. J Periodontol (2000) 71:1492–8. doi: 10.1902/jop.2000.71.9.1492 11022780

[5] Penoni DC Fidalgo TKS Torres SR Varela VM Masterson D Leão ATT. Bone density and clinical periodontal attachment in postmenopausal women: A systematic review and meta-analysis. J Dent Res (2017) 96:261–9. doi: 10.1177/0022034516682017 28048966

[6] Penoni DC Vettore MV Torres SR Farias MLF Leão ATT. An investigation of the bidirectional link between osteoporosis and periodontitis. Arch Osteoporos (2019) 14:94. doi: 10.1007/s11657-019-0643-9 31444638

[7] Xu S Zhang G Guo J Tan Y. Associations between osteoporosis and risk of periodontitis: A pooled analysis of observational studies. Oral Dis (2021) 27:357–69. doi: 10.1111/odi.13531 PMC7839743 32615008

[8] Huang H He Y-M Lin M-M Wang Y Zhang X Liang L. P2X7Rs: new therapeutic targets for osteoporosis. Purinergic Signal (2022). doi: 10.1007/s11302-021-09836-0 PMC9984661 35106736

[9] Al-Daghri NM Aziz I Yakout S Aljohani NJ Al-Saleh Y Amer OE. Inflammation as a contributing factor among postmenopausal Saudi women with osteoporosis. Med (Baltimore) (2017) 96:e5780. doi: 10.1097/MD.0000000000005780 PMC5287950 28121926

[10] De Martinis M Sirufo MM Suppa M Ginaldi L. IL-33/IL-31 axis in osteoporosis. Int J Mol Sci (2020) 21:E1239. doi: 10.3390/ijms21041239 32069819 PMC7072890

[11] Sims NA. Influences of the IL-6 cytokine family on bone structure and function. Cytokine (2021) 146:155655. doi: 10.1016/j.cyto.2021.155655 34332274

[12] Qi SS Shao ML Sun Z Chen SM Hu YJ Li XS. Chondroitin sulfate alleviates diabetic osteoporosis and repairs bone microstructure *via* anti-oxidation, anti-inflammation, and regulating bone metabolism. Front Endocrinol (Lausanne) (2021) 12:759843. doi: 10.3389/fendo.2021.759843 34777254 PMC8579055

[13] Plachokova AS Andreu-Sánchez S Noz MP Fu J Riksen NP. Oral microbiome in relation to periodontitis severity and systemic inflammation. Int J Mol Sci (2021) 22:5876. doi: 10.3390/ijms22115876 34070915 PMC8199296

[14] Bunte K Beikler T. Th17 cells and the IL-23/IL-17 axis in the pathogenesis of periodontitis and immune-mediated inflammatory diseases. Int J Mol Sci (2019) 20:3394. doi: 10.3390/ijms20143394 PMC6679067 31295952

[15] de Molon RS Rossa C Thurlings RM Cirelli JA Koenders MI. Linkage of periodontitis and rheumatoid arthritis: Current evidence and potential biological interactions. Int J Mol Sci (2019) 20:E4541. doi: 10.3390/ijms20184541 31540277 PMC6769683

[16] Czesnikiewicz-Guzik M Osmenda G Siedlinski M Nosalski R Pelka P Nowakowski D. Causal association between periodontitis and hypertension: evidence from mendelian randomization and a randomized controlled trial of non-surgical periodontal therapy. Eur Heart J (2019) 40:3459–70. doi: 10.1093/eurheartj/ehz646 PMC6837161 31504461

[17] Nwizu N Wactawski-Wende J Genco RJ. Periodontal disease and cancer: Epidemiologic studies and possible mechanisms. Periodontol 2000 (2020) 83:213–33. doi: 10.1111/prd.12329 PMC7328760 32385885

[18] Zhu M Nikolajczyk BS. Immune cells link obesity-associated type 2 diabetes and periodontitis. J Dent Res (2014) 93:346–52. doi: 10.1177/0022034513518943 PMC3957341 24393706

[19] Torrungruang K Ongphiphadhanakul B Jitpakdeebordin S Sarujikumjornwatana S. Mediation analysis of systemic inflammation on the association between periodontitis and glycaemic status. J Clin Periodontol (2018) 45:548–56. doi: 10.1111/jcpe.12884 29500831

[20] Yang J Hu S Bian Y Yao J Wang D Liu X. Targeting cell death: Pyroptosis, ferroptosis, apoptosis and necroptosis in osteoarthritis. Front Cell Dev Biol (2021) 9:789948. doi: 10.3389/fcell.2021.789948 35118075 PMC8804296

[21] Li Y Li B Liu Y Wang H He M Liu Y. Porphyromonas gingivalis lipopolysaccharide affects oral epithelial connections *via* pyroptosis. J Dent Sci (2021) 16:1255–63. doi: 10.1016/j.jds.2021.01.003 PMC8403812 34484594

[22] Yang K Xu S Zhao H Liu L Lv X Hu F. Hypoxia and porphyromonas gingivalis-lipopolysaccharide synergistically induce NLRP3 inflammasome activation in human gingival fibroblasts. Int Immunopharmacol (2021) 94:107456. doi: 10.1016/j.intimp.2021.107456 33588175

[23] Xue F Shu R Xie Y. The expression of NLRP3, NLRP1 and AIM2 in the gingival tissue of periodontitis patients: RT-PCR study and immunohistochemistry. Arch Oral Biol (2015) 60:948–58. doi: 10.1016/j.archoralbio.2015.03.005 25841070

[24] Chen Q Liu X Wang D Zheng J Chen L Xie Q. Periodontal inflammation-triggered by periodontal ligament stem cell pyroptosis exacerbates periodontitis. Front Cell Dev Biol (2021) 9:663037. doi: 10.3389/fcell.2021.663037 33869229 PMC8049442

[25] Tao Z Wang J Wen K Yao R Da W Zhou S. Pyroptosis in osteoblasts: A novel hypothesis underlying the pathogenesis of osteoporosis. Front Endocrinol (Lausanne) (2020) 11:548812. doi: 10.3389/fendo.2020.548812 33488513 PMC7821870

[26] Jiang N An J Yang K Liu J Guan C Ma C. NLRP3 inflammasome: A new target for prevention and control of osteoporosis? Front Endocrinol (Lausanne) (2021) 12:752546. doi: 10.3389/fendo.2021.752546 34646239 PMC8502943

[27] Alippe Y Mbalaviele G. Omnipresence of inflammasome activities in inflammatory bone diseases. Semin Immunopathol (2019) 41:607–18. doi: 10.1007/s00281-019-00753-4 PMC6814643 31520179

[28] Zhou Y Zhou B Pache L Chang M Khodabakhshi AH Tanaseichuk O. Metascape provides a biologist-oriented resource for the analysis of systems-level datasets. Nat Commun (2019) 10:1523. doi: 10.1038/s41467-019-09234-6 30944313 PMC6447622

[29] Zhuang Z Cai H Lin H Guan B Wu Y Zhang Y. Development and validation of a robust pyroptosis-related signature for predicting prognosis and immune status in patients with colon cancer. J Oncol (2021) 2021:5818512. doi: 10.1155/2021/5818512 34840571 PMC8616665

[30] Ye Y Dai Q Qi H. A novel defined pyroptosis-related gene signature for predicting the prognosis of ovarian cancer. Cell Death Discovery (2021) 7:71. doi: 10.1038/s41420-021-00451-x 33828074 PMC8026591

[31] Luo P Jiang Y Ding S Jiang S Tang R Tang Z. Integrative analysis of pyroptosis-related prognostic signature and immunological infiltration in lung squamous cell carcinoma. BioMed Res Int (2022) 2022:4944758. doi: 10.1155/2022/4944758 35692583 PMC9177339

[32] Wu J Zhu Y Luo M Li L. Comprehensive analysis of pyroptosis-related genes and tumor microenvironment infiltration characterization in breast cancer. Front Immunol (2021) 12:748221. doi: 10.3389/fimmu.2021.748221 34659246 PMC8515898

[33] Akoglu H. User’s guide to correlation coefficients. Turk J Emerg Med (2018) 18:91–3. doi: 10.1016/j.tjem.2018.08.001 PMC6107969 30191186

[34] Kanehisa M Goto S. KEGG: kyoto encyclopedia of genes and genomes. Nucleic Acids Res (2000) 28:27–30. doi: 10.1093/nar/28.1.27 10592173 PMC102409

[35] Shannon P. Cytoscape: a software environment for integrated models of biomolecular interaction networks’. Genome Res (2003) 13:2498–504. doi: 10.1101/gr.1239303 PMC403769 14597658

[36] Du Y Cai M Xing X Ji J Yang E Wu J. PINA 3.0: mining cancer interactome. Nucleic Acids Res (2021) 49:D1351–7. doi: 10.1093/nar/gkaa1075 PMC7779002 33231689

[37] UniProt Consortium. UniProt: the universal protein knowledgebase in 2021. Nucleic Acids Res (2021) 49:D480–9. doi: 10.1093/nar/gkaa1100 PMC7778908 33237286

[38] Szklarczyk D Gable AL Nastou KC Lyon D Kirsch R Pyysalo S. The STRING database in 2021: customizable protein-protein networks, and functional characterization of user-uploaded gene/measurement sets. Nucleic Acids Res (2021) 49:D605–12. doi: 10.1093/nar/gkaa1074 PMC7779004 33237311

[39] Rahiminejad S Maurya MR Subramaniam S. The community structure of the network is interpreted as the spin configuration that minimizes the energy of the spinglass. BMC Bioinf (2019) 20:212. doi: 10.1186/s12859-019-2746-0

[40] Smith NR Zivich PN Frerichs LM Moody J Aiello AE. A guide for choosing community detection algorithms in social network studies: The question alignment approach. Am J Prev Med (2020) 59:597–605. doi: 10.1016/j.amepre.2020.04.015 32951683 PMC7508227

[41] Chin C-H Chen S-H Wu H-H Ho C-W Ko M-T Lin C-Y. cytoHubba: identifying hub objects and sub-networks from complex interactome. BMC Syst Biol (2014) 8 Suppl 4:S11. doi: 10.1186/1752-0509-8-S4-S11 25521941 PMC4290687

[42] Nahm FS. Receiver operating characteristic curve: overview and practical use for clinicians. Korean J Anesthesiol (2022) 75:25–36. doi: 10.4097/kja.21209 35124947 PMC8831439

[43] Carbon S Ireland A Mungall CJ Shu S Marshall B Lewis S. AmiGO: online access to ontology and annotation data. Bioinformatics (2009) 25:288–9. doi: 10.1093/bioinformatics/btn615 PMC2639003 19033274

[44] Bindea G Mlecnik B Hackl H Charoentong P Tosolini M Kirilovsky A. ClueGO: a cytoscape plug-in to decipher functionally grouped gene ontology and pathway annotation networks. Bioinformatics (2009) 25:1091–3. doi: 10.1093/bioinformatics/btp101 PMC2666812 19237447

[45] Newman AM Liu CL Green MR Gentles AJ Feng W Xu Y. Robust enumeration of cell subsets from tissue expression profiles. Nat Methods (2015) 12:453–7. doi: 10.1038/nmeth.3337 PMC4739640 25822800

[46] Wang Y Kanneganti T-D. From pyroptosis, apoptosis and necroptosis to PANoptosis: A mechanistic compendium of programmed cell death pathways. Comput Struct Biotechnol J (2021) 19:4641–57. doi: 10.1016/j.csbj.2021.07.038 PMC8405902 34504660

[47] Potts BB. Network Analysis: A handbook/social network analysis: methods and applications (book). Acta Sociol (1994) 37:419–23. (Taylor & Francis Ltd).

[48] Li W Yin Y Quan X Zhang H. Gene expression value prediction based on XGBoost algorithm. Front Genet (2019) 10:1077. doi: 10.3389/fgene.2019.01077 31781160 PMC6861218

[49] Holmstrup P Damgaard C Olsen I Klinge B Flyvbjerg A Nielsen CH. Comorbidity of periodontal disease: two sides of the same coin? an introduction for the clinician. J Oral Microbiol (2017) 9:1332710. doi: 10.1080/20002297.2017.1332710 28748036 PMC5508374

[50] Yu B Wang C-Y. Osteoporosis and periodontal diseases - an update on their association and mechanistic links. Periodontol 2000 (2022) 89:99–113. doi: 10.1111/prd.12422 PMC9067601 35244945

[51] Hajishengallis G Chavakis T. Local and systemic mechanisms linking periodontal disease and inflammatory comorbidities. Nat Rev Immunol (2021) 21:426–40. doi: 10.1038/s41577-020-00488-6 PMC7841384 33510490

[52] Yu P Zhang X Liu N Tang L Peng C Chen X. Pyroptosis: mechanisms and diseases. Signal Transduct Target Ther (2021) 6:128. doi: 10.1038/s41392-021-00507-5 33776057 PMC8005494

[53] Alam MI Mae M Farhana F Oohira M Yamashita Y Ozaki Y. NLRP3 inflammasome negatively regulates RANKL-induced osteoclastogenesis of mouse bone marrow macrophages but positively regulates it in the presence of lipopolysaccharides. Int J Mol Sci (2022) 23:6096. doi: 10.3390/ijms23116096 35682777 PMC9181162

[54] Jiang M Shang Z Zhang T Yin X Liang X Sun H. Study on the role of pyroptosis in bone resorption induced by occlusal trauma with or without periodontitis. J Periodontal Res (2022) 57:448–60. doi: 10.1111/jre.12974 35141913

[55] Zhu X Zhang K Lu K Shi T Shen S Chen X. Inhibition of pyroptosis attenuates staphylococcus aureus-induced bone injury in traumatic osteomyelitis. Ann Transl Med (2019) 7:170. doi: 10.21037/atm.2019.03.40 31168451 PMC6526268

[56] Li Y Ling J Jiang Q. Inflammasomes in alveolar bone loss. Front Immunol (2021) 12:691013. doi: 10.3389/fimmu.2021.691013 34177950 PMC8221428

[57] Sordi MB Magini R de S Panahipour L Gruber R. Pyroptosis-mediated periodontal disease. Int J Mol Sci (2021) 23:372. doi: 10.3390/ijms23010372 35008798 PMC8745163

[58] Tao H Li W Zhang W Yang C Zhang C Liang X. Urolithin a suppresses RANKL-induced osteoclastogenesis and postmenopausal osteoporosis by, suppresses inflammation and downstream NF-κB activated pyroptosis pathways. Pharmacol Res (2021) 174:105967. doi: 10.1016/j.phrs.2021.105967 34740817

[59] Van Opdenbosch N Lamkanfi M. Caspases in cell death, inflammation, and disease. Immunity (2019) 50:1352–64. doi: 10.1016/j.immuni.2019.05.020 PMC6611727 31216460

[60] Rana AK Li Y Dang Q Yang F. Monocytes in rheumatoid arthritis: Circulating precursors of macrophages and osteoclasts and, their heterogeneity and plasticity role in RA pathogenesis. Int Immunopharmacol (2018) 65:348–59. doi: 10.1016/j.intimp.2018.10.016 30366278

[61] Greenhill CJ Jones GW Nowell MA Newton Z Harvey AK Moideen AN. Interleukin-10 regulates the inflammasome-driven augmentation of inflammatory arthritis and joint destruction. Arthritis Res Ther (2014) 16:419. doi: 10.1186/s13075-014-0419-y 25175678 PMC4292830

[62] Kawano T Inokuchi J Eto M Murata M Kang J-H. Activators and inhibitors of protein kinase c (PKC): Their applications in clinical trials. Pharmaceutics (2021) 13:1748. doi: 10.3390/pharmaceutics13111748 34834162 PMC8621927

[63] Hagel-Bradway S Dziak R. Regulation of bone cell metabolism. J Oral Pathol Med (1989) 18:344–51. doi: 10.1111/j.1600-0714.1989.tb01564.x 2681687

[64] Shin J Jang H Lin J Lee SY. PKCβ positively regulates RANKL-induced osteoclastogenesis by inactivating GSK-3β. Mol Cells (2014) 37:747–52. doi: 10.14348/molcells.2014.0220 PMC4213766 25256217

[65] Yao J Li J Zhou L Cheng J Chim SM Zhang G. Protein kinase c inhibitor, GF109203X attenuates osteoclastogenesis, bone resorption and RANKL-induced NF-κB and NFAT activity. J Cell Physiol (2015) 230:1235–42. doi: 10.1002/jcp.24858 25363829

[66] Lin R Taylor BV Simpson S Charlesworth J Ponsonby A-L Pittas F. Novel modulating effects of PKC family genes on the relationship between serum vitamin d and relapse in multiple sclerosis. J Neurol Neurosurg Psychiatry (2014) 85:399–404. doi: 10.1136/jnnp-2013-305245 23868949

[67] Shi J Zhao Y Wang K Shi X Wang Y Huang H. Cleavage of GSDMD by inflammatory caspases determines pyroptotic cell death. Nature (2015) 526:660–5. doi: 10.1038/nature15514 26375003

[68] Wang K Sun Q Zhong X Zeng M Zeng H Shi X. Structural mechanism for GSDMD targeting by autoprocessed caspases in pyroptosis. Cell (2020) 180:941–955.e20. doi: 10.1016/j.cell.2020.02.002 32109412

[69] Sun K Wang C Xiao J Brodt MD Yuan L Yang T. Fracture healing is delayed in the absence of gasdermin-interleukin-1 signaling. Elife (2022) 11:e75753. doi: 10.7554/eLife.75753 35244027 PMC8923664

[70] Xiao J Wang C Yao J-C Alippe Y Yang T Kress D. Radiation causes tissue damage by dysregulating inflammasome-gasdermin d signaling in both host and transplanted cells. PloS Biol (2020) 18:e3000807. doi: 10.1371/journal.pbio.3000807 32760056 PMC7446913

[71] Yang L Liu J Shan Q Geng G Shao P. High glucose inhibits proliferation and differentiation of osteoblast in alveolar bone by inducing pyroptosis. Biochem Biophys Res Commun (2020) 522:471–8. doi: 10.1016/j.bbrc.2019.11.080 31780258

[72] Rathkey JK Xiao TS Abbott DW. Human polymorphisms in GSDMD alter the inflammatory response. J Biol Chem (2020) 295:3228–38. doi: 10.1074/jbc.RA119.010604 PMC7062166 31988247

[73] Kesavardhana S Malireddi RKS Kanneganti T-D. Caspases in cell death, inflammation, and pyroptosis. Annu Rev Immunol (2020) 38:567–95. doi: 10.1146/annurev-immunol-073119-095439 PMC7190443 32017655

[74] McIlwain DR Berger T Mak TW. Caspase functions in cell death and disease. Cold Spring Harb Perspect Biol (2013) 5:a008656. doi: 10.1101/cshperspect.a008656 23545416 PMC3683896

[75] Yan S Li YZ Zhu XW Liu CL Wang P Liu YL. HuGE systematic review and meta-analysis demonstrate association of CASP-3 and CASP-7 genetic polymorphisms with cancer risk. Genet Mol Res (2013) 12:1561–73. doi: 10.4238/2013.May.13.10 23765963

[76] Mori G Brunetti G Colucci S Oranger A Ciccolella F Sardone F. Osteoblast apoptosis in periodontal disease: role of TNF-related apoptosis-inducing ligand. Int J Immunopathol Pharmacol (2009) 22:95–103. doi: 10.1177/039463200902200111 19309556

[77] Bantel H Beikler T Flemmig TF Schulze-Osthoff K. Caspase activation is involved in chronic periodontitis. FEBS Lett (2005) 579:5559–64. doi: 10.1016/j.febslet.2005.09.020 16213496

[78] Liu H Wang Y-W Chen W-D Dong H-H Xu Y-J. Iron accumulation regulates osteoblast apoptosis through lncRNA XIST/miR-758-3p/caspase 3 axis leading to osteoporosis. IUBMB Life (2021) 73:432–43. doi: 10.1002/iub.2440 33336851

[79] Jiang M Qi L Li L Li Y. The caspase-3/GSDME signal pathway as a switch between apoptosis and pyroptosis in cancer. Cell Death Discovery (2020) 6:112. doi: 10.1038/s41420-020-00349-0 33133646 PMC7595122

[80] Kang SW Kim SK Chung JH Ban JY. Assessment of CASP gene polymorphisms in periodontal disease. Genet Mol Res (2015) 14:18069–77. doi: 10.4238/2015.December.22.33 26782454

[81] López-Doménech G Serrat R Mirra S D’Aniello S Somorjai I Abad A. The eutherian armcx genes regulate mitochondrial trafficking in neurons and interact with miro and Trak2. Nat Commun (2012) 3:814. doi: 10.1038/ncomms1829 22569362

[82] Wang T Zhong H Qin Y Wei W Li Z Huang M. ARMCX family gene expression analysis and potential prognostic biomarkers for prediction of clinical outcome in patients with gastric carcinoma. BioMed Res Int (2020) 2020:3575038. doi: 10.1155/2020/3575038 32685472 PMC7345962

[83] Mirra S Gavaldà-Navarro A Manso Y Higuera M Serrat R Salcedo MT. ARMCX3 mediates susceptibility to hepatic tumorigenesis promoted by dietary lipotoxicity. Cancers (Basel) (2021) 13:1110. doi: 10.3390/cancers13051110 33807672 PMC7961652

[84] Du J Zhang X Zhou H Miao Y Han Y Han Q. Alex3 suppresses non-small cell lung cancer invasion *via* AKT/Slug/E-cadherin pathway. Tumour Biol (2017) 39:1010428317701441. doi: 10.1177/1010428317701441 28705116

[85] Meinhardt G Roth J Hass R. Activation of protein kinase c relays distinct signaling pathways in the same cell type: differentiation and caspase-mediated apoptosis. Cell Death Differ (2000) 7:795–803. doi: 10.1038/sj.cdd.4400709 11042674

[86] Rogers C Fernandes-Alnemri T Mayes L Alnemri D Cingolani G Alnemri ES. Cleavage of DFNA5 by caspase-3 during apoptosis mediates progression to secondary necrotic/pyroptotic cell death. Nat Commun (2017) 8:1–14. doi: 10.1038/ncomms14128 28045099 PMC5216131

[87] Rodríguez-Carballo E Gámez B Ventura F. p38 MAPK signaling in osteoblast differentiation. Front Cell Dev Biol (2016) 4:40. doi: 10.3389/fcell.2016.00040 27200351 PMC4858538

[88] Yong H-Y Koh M-S Moon A. The p38 MAPK inhibitors for the treatment of inflammatory diseases and cancer. Expert Opin Investig Drugs (2009) 18:1893–905. doi: 10.1517/13543780903321490 19852565

[89] Keshari RS Verma A Barthwal MK Dikshit M. Reactive oxygen species-induced activation of ERK and p38 MAPK mediates PMA-induced NETs release from human neutrophils. J Cell Biochem (2013) 114:532–40. doi: 10.1002/jcb.24391 22961925

[90] Wang Y Wang W Wang N Tall AR Tabas I. Mitochondrial oxidative stress promotes atherosclerosis and neutrophil extracellular traps in aged mice. Arteriosc Thromb Vasc Biol (2017) 37:e99–e107. doi: 10.1161/ATVBAHA.117.309580 PMC5535797 28596373

[91] Wang J Zhou Y Ren B Zou L He B Li M. The role of neutrophil extracellular traps in periodontitis. Front Cell Infect Microbiol (2021) 11:639144. doi: 10.3389/fcimb.2021.639144 33816343 PMC8012762

[92] Lopes DEM Jabr CL Dejani NN Saraiva AC de Aquino SG Medeiros AI. Inhibition of 5-Lipoxygenase (5-Lo) attenuates inflammation and bone resorption in lipopolysaccharide (Lps)-Induced Periodontal Disease. J Periodontology (2017) 89:235–45. doi: 10.1902/jop.2017.170210 28871891

[93] Oliveira SR de Arruda JAA Schneider AH Carvalho VF Machado CC Corrêa JD. Are neutrophil extracellular traps the link for the cross-talk between periodontitis and rheumatoid arthritis physiopathology? Rheumatol (Oxford) (2021) 61:174–84. doi: 10.1093/rheumatology/keab289 33752229

[94] Kurtz JR Goggins JA McLachlan JB. Salmonella infection: Interplay between the bacteria and host immune system. Immunol Lett (2017) 190:42–50. doi: 10.1016/j.imlet.2017.07.006 28720334 PMC5918639

[95] Chang M-L Liaw Y-F. Hepatitis b flare in hepatitis b e antigen-negative patients: A complicated cascade of innate and adaptive immune responses. Int J Mol Sci (2022) 23:1552. doi: 10.3390/ijms23031552 35163476 PMC8836007

[96] Nüssing S Sant S Koutsakos M Subbarao K Nguyen THO Kedzierska K. Innate and adaptive T cells in influenza disease. Front Med (2018) 12:34–47. doi: 10.1007/s11684-017-0606-8 29352371

[97] Towers GJ Noursadeghi M. Interactions between HIV-1 and the cell-autonomous innate immune system. Cell Host Microbe (2014) 16:10–8. doi: 10.1016/j.chom.2014.06.009 PMC4096382 25011104

[98] Wu Z Qin R Wang L Bosso M Scherer M Stamminger T. Human cytomegalovirus particles treated with specific antibodies induce intrinsic and adaptive but not innate immune responses. J Virol (2017) 91:e00678–17. doi: 10.1128/JVI.00678-17 PMC5660505 28878085

[99] McClellan K Howard K Mayhew E Niederkorn J Alizadeh H. Adaptive immune responses to acanthamoeba cysts. Exp Eye Res (2002) 75:285–93. doi: 10.1006/exer.2002.2015 12384091

[100] Cekici A Kantarci A Hasturk H Van Dyke TE. Inflammatory and immune pathways in the pathogenesis of periodontal disease. Periodontol 2000 (2014) 64:57–80. doi: 10.1111/prd.12002 24320956 PMC4500791

[101] Hajishengallis G. Periodontitis: from microbial immune subversion to systemic inflammation. Nat Rev Immunol (2015) 15:30–44. doi: 10.1038/nri3785 25534621 PMC4276050

[102] Schenkein HA Papapanou PN Genco R Sanz M. Mechanisms underlying the association between periodontitis and atherosclerotic disease. Periodontol 2000 (2020) 83:90–106. doi: 10.1111/prd.12304 32385879

[103] Fischer V Haffner-Luntzer M. Interaction between bone and immune cells: Implications for postmenopausal osteoporosis. Semin Cell Dev Biol (2022) 123:14–21. doi: 10.1016/j.semcdb.2021.05.014 34024716

[104] Saxena Y Routh S Mukhopadhaya A. Immunoporosis: Role of innate immune cells in osteoporosis. Front Immunol (2021) 12:687037. doi: 10.3389/fimmu.2021.687037 34421899 PMC8374941

[105] Irwandi RA Kuswandani SO Harden S Marletta D D’Aiuto F. Circulating inflammatory cell profiling and periodontitis: A systematic review and meta-analysis. J Leukoc Biol (2022) 111:1069–96. doi: 10.1002/JLB.5RU1021-524R 35199874

[106] Li Y Toraldo G Li A Yang X Zhang H Qian W-P. B cells and T cells are critical for the preservation of bone homeostasis and attainment of peak bone mass *in vivo* . Blood (2007) 109:3839–48. doi: 10.1182/blood-2006-07-037994 PMC1874582 17202317

[107] Li W Zhang Z Wang Z-M. Differential immune cell infiltrations between healthy periodontal and chronic periodontitis tissues. BMC Oral Health (2020) 20:293. doi: 10.1186/s12903-020-01287-0 33109155 PMC7590666

[108] Weitzmann MN. Bone and the immune system. Toxicol Pathol (2017) 45:911–24. doi: 10.1177/0192623317735316 PMC5749254 29046115

[109] Wang W Liu H Liu T Yang H He F. Insights into the role of macrophage polarization in the pathogenesis of osteoporosis. Oxid Med Cell Longev (2022) 2022:2485959. doi: 10.1155/2022/2485959 35707276 PMC9192196

[110] Muñoz J Akhavan NS Mullins AP Arjmandi BH. Macrophage polarization and osteoporosis: A review. Nutrients (2020) 12:E2999. doi: 10.3390/nu12102999 33007863 PMC7601854

