# Supplementary figures and images for: Screening of crosstalk and pyroptosis-related genes linking periodontitis and osteoporosis based on bioinformatics and machine learning
Source: Front Immunol. 2022 Aug 5;13:955441. doi: 10.3389/fimmu.2022.955441 (PMC9389017; doi:10.3389/fimmu.2022.955441)

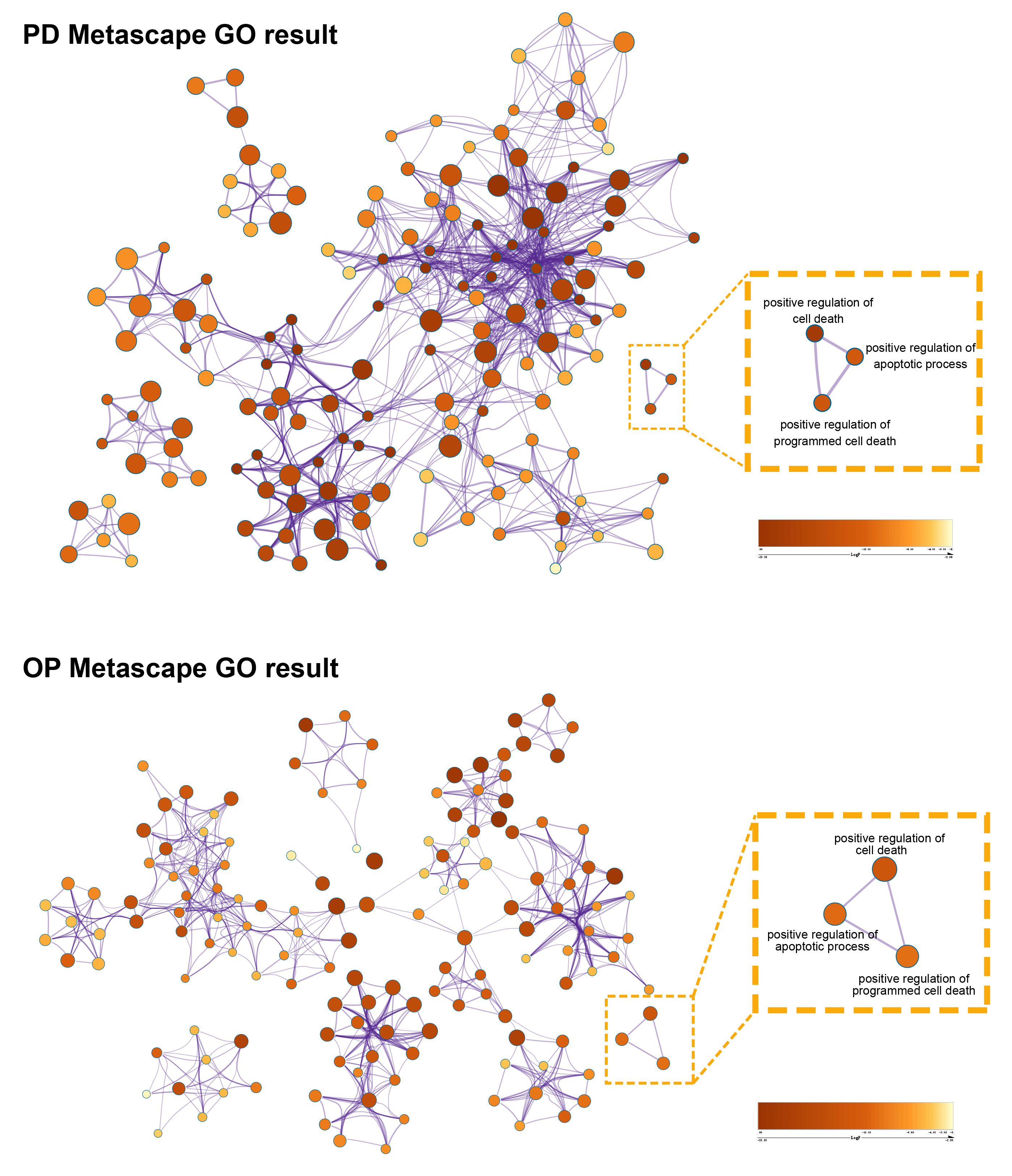

Supplement: Supplementary Figure 1 — The GO clustering results of Metascape. [file Image_1.jpeg]
